# The GENEVA platform models tumor mosaicism to reveal variations of responses to KRAS inhibitors and identify improved drug combinations

**DOI:** 10.1038/s43018-026-01130-5

**Published:** 2026-02-24

**Authors:** Johnny X. Yu, Jung Min Suh, Katerina D. Popova, Kristle Garcia, Tanvi Joshi, Bruce Culbertson, Jessica B. Spinelli, Vishvak Subramanyam, Kevin Lou, Trey Charbonneau, Kevan M. Shokat, Jonathan Weissman, Hani Goodarzi

**Affiliations:** 1 Department of Biochemistry and Biophysics, University of California, San Francisco, San Francisco, CA USA; 2 Department of Urology, University of California, San Francisco, San Francisco, CA USA; 3 Helen Diller Family Comprehensive Cancer Center, University of California, San Francisco, San Francisco, CA USA; 4 Bakar Computational Health Sciences Institute, University of California, San Francisco, San Francisco, CA USA; 5 Biomedical Sciences Graduate Program, University of California, San Francsicso, San Francisco, CA USA; 6 Arc Institute, Palo Alto, CA USA; 7 Department of Biology, Whitehead Institute, MIT, Cambridge, MA USA; 8 Department of Cellular and Molecular Pharmacology, University of California, San Francisco, San Francisco, CA USA; 9 Program in Molecular Medicine, University of Massachusetts Chan Medical School, Worcester, MA USA; 10 Howard Hughes Medical Institute, Bethesda, MD USA

**Keywords:** Cancer, Cancer therapy, Cancer models, Cancer genomics

## Abstract

The clinical success of cancer drug candidates depends on efficacy across many different individuals. Because xenografts are challenging to scale, we currently rely on a limited set of in vivo preclinical models. Here, to address this limitation, we introduce GENEVA, a scalable single-cell-resolution platform for measuring responses to drug perturbations. GENEVA models cancer genetic diversity by combining multiple patient-derived cell lines and cancer cell lines into pooled three-dimensional cultures and xenograft models, allowing us to study drug responses across a wide range of genetic backgrounds within a single experiment. We apply GENEVA to investigate KRAS-G12C inhibitors and demonstrate that mitochondrial activation is a key driver of cell death following KRAS inhibition, while epithelial-to-mesenchymal transition is a prominent resistance mechanism. These findings highlight the utility of GENEVA to identify therapeutic targets and optimize combination therapies with the potential to bridge the gap between preclinical cancer models and patient outcomes.

## Abstract (web summary)

Yu et al. develop a platform to model genetic diversity in drug treatment at the single-cell level. Using KRAS-G12C inhibitors as a proof of concept, they identify the pathway driving cell death and potential resistance mechanisms.

## Main

Targeted therapies aim to precisely attack cancer cells by focusing on their molecular and genetic drivers^1^. However, even with these advances, variations between tumor responses make it challenging to develop drug candidates across a range of patient models^2^. Although in vivo xenografts are the gold standard of disease models used in late-stage preclinical development, they are costly and labor intensive. This lack of scalability limits the ability to test drugs in large cohorts of models before proceeding to clinical trials where success is determined by efficacy across many diverse individuals^3^. In addition, the lack of scalability makes it prohibitive to apply the latest single-cell genomic technologies, which allow high-resolution profiling of clinical samples and chemical perturbation^4,5^, to early drug discovery pipelines^4,6^.

To address this core issue, we introduce GENetically diverse and Endogenously controlled phenotypic Variation Assay (GENEVA), a framework enabling scalable molecular phenotyping of multiplexed tumor models in two-dimensional/three-dimensional (2D/3D) culture and xenografted mice. GENEVA uses high-resolution molecular data from various cancer cell models to capture inter- and intrapopulation heterogeneity in drug response at substantially longer time points than prior methods. By studying drug effects across genotypes within an internally controlled setting, GENEVA uncovers generalizable consequences of drug action across tumors.

We applied GENEVA to study first-in-class RAS inhibitors approved in 2021, specifically compounds targeting the oncogenic *KRAS*^G12C^ mutation^7,8^. Although these compounds recently invigorated the field with their rapid development and clinical approval, several key challenges and gaps in knowledge remain. Notably, the first approved KRAS-G12C inhibitor sotorasib has a median progression-free survival of only 6 months, and multiple resistance mechanisms develop in individuals with refractory disease^9,10^. By creating mosaic populations of *KRAS* variants (G12C and non-G12C) in vitro and in vivo, we revealed insights into the biology of these RAS inhibitors: (1) a role for mitochondrial activity downstream of RAS inhibition causing cell death, (2) implication of multiple pathways in KRAS-G12C inhibitor tolerance and (3) identification of resistance mechanisms that emerge strongly in vivo. In addition to showcasing the power of GENEVA in understanding the broader biological impact of chemical perturbations, these findings lay the groundwork for advancing the emerging class of targeted RAS inhibitors and expanding combination therapies that address these diverse biological mechanisms.

## Results

### Multiplexed rich phenotyping to identify determinants of drug response and tolerance

In a GENEVA experiment, cells from tens of genetic backgrounds are combined into a single pool that is then grown in 3D cultures or xenografted in mice. The resulting mosaic tumor model serves as the unit of observation and is subjected to chemical perturbations in vivo or in vitro. The pool then undergoes single-cell profiling, and the identity of each cell is determined based on known reference genotypes. This approach simultaneously captures macroscopic, cellular and molecular phenotypes across patient-derived lines.

**Fig. 1:**
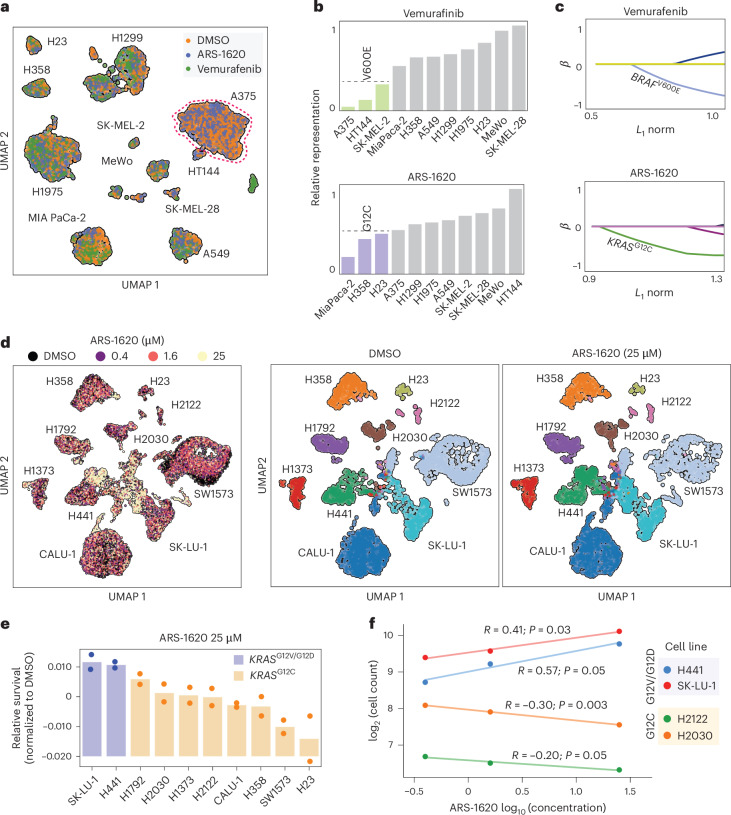
GENEVA allows for rich phenotypic profiling of cells in genetically diverse populations. **a**, Our GENEVA pilot study with 11 human cell lines and three treatment conditions: DMSO, ARS-1620 (1 µM) and vemurafenib (0.8 µM) treatment for 6 days. The uniform manifold approximation and projection (UMAP) summarizes the resulting scRNA-seq data. Cells are colored based on their drug treatment, and the cell line identity of each cluster is labeled. The A375 cluster is marked by a dashed red circle. **b**, The fraction of cells in each cell line in the treatment versus control populations was used to quantitatively measure the sensitivity of each cell line. Cell counts are from the genotyped scRNA-seq data. Lines are sorted based on their relative sensitivity, and G12C and V600E mutant lines are marked in ARS-1620 and vemurafenib plots, respectively. Relative representation was calculated by normalizing the cell counts by cell line by the maximum number of cells in each respective drug condition; this ratio is shown. **c**, Lasso regression with overlapping genetic mutations as covariates and GENEVA relative survival data as outcomes. Variables showing selection by the model are colored and labeled. *KRAS*^G12C^ and *BRAF*^V600E^ mutations are marked in each plot. **d**, Summary of a G12C GENEVA pool composed of eight G12C cell lines along with two non-G12C lines (G12V and G12D) and three doses of ARS-1620 administered for 6 days. Shown are the UMAPs for the DMSO and 25 μM ARS-1620 conditions, respectively. **e**, Cell line representation at the highest dose relative to DMSO was used to measure relative drug sensitivity for each sample. Results are shown as a sorted bar graph where individual biological replicates are included as points with the bar showing the mean of the data (*n* = 2). **f**, Comparison of cell representation across doses for two G12C and two non-G12C lines. Lines are colored by identity and grouped into G12C and non-G12C lines, with G12C lines showing negative slopes across dose titrations of ARS-1620 (*n* = 2 biological replicates for all doses, where each point is the average). Statistical significance was assessed using a one-sided test for significance of Pearson’s *R*. Source data

**Extended Data Fig. 1:**
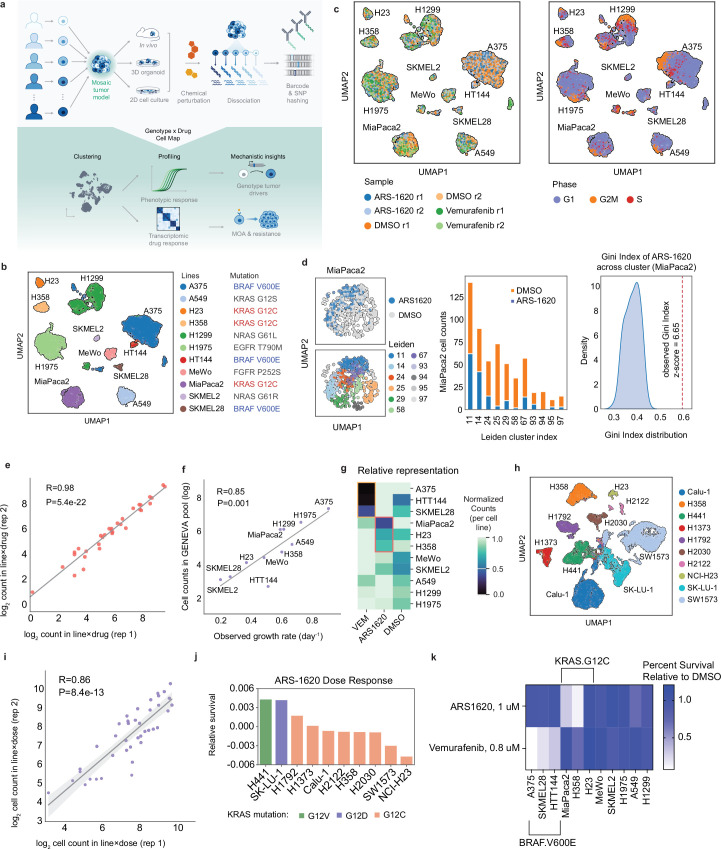
GENEVA schematic and experimental results. **(a)** Conceptual overview of GENEVA as a multiplexed method for rich phenotyping of diverse populations. Mechanisms of drug efficacy and escape are elucidated using a mosaic tumor model approach. **(b)** UMAP projection of a GENEVA pool composed of heterogeneous cell lines from different genetic driver backgrounds. Clusters are colored by cell line identity, **(c)** drug treatment condition, and cell cycle phase. **(d)** UMAP projection of *KRAS* G12C mutant cell line MIA PaCa-2 colored by drug treatment condition and Leiden sub-clustering (left). Cells binned by cluster representation across drug treatment and vehicle groups (center). Gini index distribution compared for drug and vehicle conditions across clusters (right). **(e)** Log_2_ of cell counts across two independent replicates showing concordance of cell counts after mosaic pool growth. **(f)** Comparison of Log_2_ cell counts observed with growth rates measured in vitro. **(g)** Heatmap of relative drug sensitivity in treatment conditions normalized on a per cell line basis from GENEVA cell counts. Dark blue and white indicate lower and higher relative representation respectively as denoted by the legend which displays the cell counts, normalized within each cell line, in different treatment conditions. **(h)** UMAP projection of a GENEVA pool with a cell line composition focused on *KRAS* G12* mutant alleles. Clusters are colored by cell line identity. **(i)** Log_2_ of cell counts from focused *KRAS* G12* pool across two independent replicates. **(j)** Relative survival of cell pools normalized to vehicle pools for each cell line. Cell lines are colored by the identity of *KRAS* G12* mutation. **(k)** Relative survival as measured by cell counts of drug treatments across different cancer models in traditional cellular growth inhibition assays over six days. Statistical significance of correlation assessed by two-sided Pearson correlation test for (**e**), (**f**), and (**i**). Source data

For an initial test, we pooled 11 cell lines, including ones with *BRAF*^V600E^ and *KRAS*^G12C^ mutations known for their sensitivity to the mutation-specific inhibitors vemurafenib and ARS-1620, respectively^11–14^. We split this pool into DMSO, vemurafenib and ARS-1620 conditions and used MULTI-seq reagents to hash individual samples and combine them into one single-cell RNA sequencing (scRNA-seq) run^15^. Next, we used single-nucleotide polymorphism-based deconvolution and hash oligonucleotide demultiplexing to determine the genetic identity and treatment condition for each profiled cell (Fig. 1a and Extended Data Fig. 1a–c)^16^. We noted several expected patterns, such as A375 cells (*BRAF*^V600E^ mutant; red dashed outline in Fig. 1a) being highly depleted by vemurafenib treatment^17^ and MIA PaCa-2 cells (*KRAS*^G12C^ mutant) being highly depleted by ARS-1620 treatment (Extended Data Fig. 1d)^18^. We also observed intrapopulation heterogeneity in the sensitivity of MIA PaCa-2 cells to ARS-1620, where the distribution of ARS-1620-treated MIA PaCa-2 cells across the possible gene expression landscape significantly deviates from random chance (*z* score of 6.65; Extended Data Fig. 1d). These observations show that both the number and transcriptomic state of cells in GENEVA effectively capture phenotypic traits such as sensitivity and response heterogeneity (Extended Data Fig. 1e). Cell counts were highly reproducible between biological replicates for all cell line–drug combinations. Furthermore, we observed an expected correlation between relative cell counts and growth rates in the control sample, which validated the line-to-line seeding ratios we had characterized and used to balance cell line representation in GENEVA pools after multiple days of growth (Extended Data Fig. 1f).

We then further analyzed the relationship between cell line representation and drug sensitivity. As expected, *BRAF*^V600E^ mutants were most sensitive to vemurafenib, and *KRAS*^G12C^ mutants were more sensitive to ARS-1620 (Fig. 1b and Extended Data Fig. 1g). Lasso regression analysis, with GENEVA-based relative sensitivities as response and whole-exome-derived somatic mutations as covariates, correctly captured *BRAF*^V600E^ and *KRAS*^G12C^ as drivers of drug sensitivity to vemurafenib and ARS-1620, respectively (Fig. 1c).

Next, to better understand the molecular basis for inter- and intraline variations in ARS-1620 response among different *KRAS* variant lines, we conducted a focused study using a pool of eight G12C and two non-G12C lung cancer cell lines. We treated these GENEVA pools with three concentrations of ARS-1620 for 6 days, performed scRNA-seq and analyzed the genotype of each cell as described earlier (Fig. 1d and Extended Data Fig. 1h). We again observed highly reproducible cell counts for two replicates across treatments and cell lines (*R* = 0.9; Extended Data Fig. 1i) and expected G12C-dependent changes in pool representation, with non-G12C lines becoming relatively more abundant at higher ARS-1620 concentrations (Fig. 1e). Furthermore, akin to a dose–response analysis, the multiple concentrations of ARS-1620 allowed us to assess changes in pool representation as a function of drug concentration. For example, the relative pool representation of non-G12C lines (H441 and SK-LU-1 cells) increased across ARS-1620 concentrations, whereas G12C lines (H2122 and H2030) decreased (Fig. 1f). We calculated a sensitivity score for each line by measuring the slope in the log–log plots presented and observed a clear demarcation between G12C and non-G12C lines as well as a range of sensitivity among the *KRAS*^G12C^ mutant backgrounds (Extended Data Fig. 1j). These sensitivity patterns showed concordance with in vitro growth inhibition assays we had performed with monocultures of these same cell lines (Extended Data Fig. 1k).

### In vivo GENEVA for multiplexed high-content phenotyping in xenograft models

**Fig. 2:**
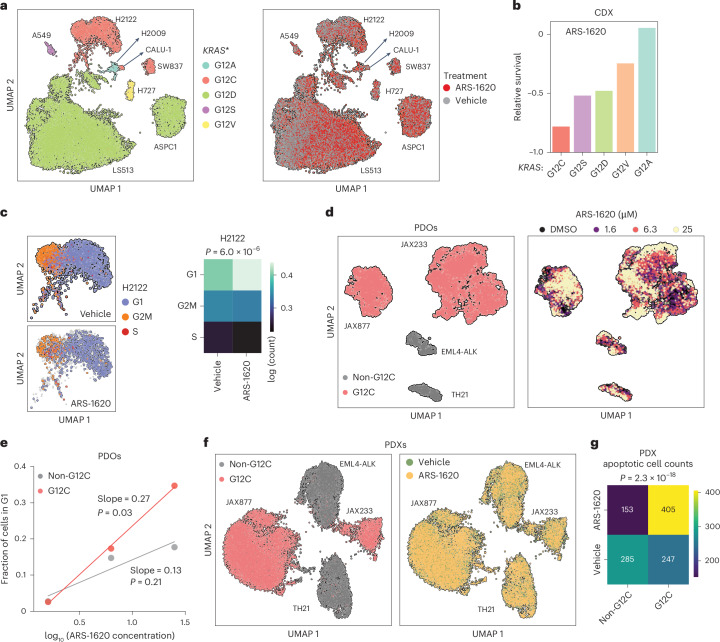
In vivo GENEVA applied to *KRAS*^G12*^ CDX and PDX models. **a**, NOD.Cg-*Prkdc*^scid^Il2rg^tm1Wjl^/SzJ (NSG) mice xenografted with mosaic cell line models from a pool of *KRAS*^G12*^ mutants were treated with ARS-1620 (100 mg per kg (body weight)) or vehicle control for 10 days after injection. Individual cells are colored by either *KRAS*^G12*^ mutant status (left) or the treatment condition (right). **b**, In vivo sensitivity phenotypes from CDX GENEVA mosaic tumors. Relative survival scores were calculated for each cell line by comparing their counts in drug and vehicle conditions and aggregating across different *KRAS*^G12*^ mutations. **c**, UMAP of H2122 cells in the CDX GENEVA dataset colored by cell cycle phase (left). The number of cells in each phase was tabulated for ARS-1620- and vehicle-treated samples (right). The *P* value was calculated using a two-sided *χ*^2^ test. **d**, UMAP of mosaic PDOs in an ARS-1620 GENEVA experiment. The cells are colored by G12C status (left) and ARS-1620 dose (right). Organoids were treated for 96 h. **e**, Relationship between the fraction of cells in G1 and ARS-1620 concentration for G12C (red) and non-G12C PDOs (*n* = 1 for all doses). Shown are the slopes and their associated two-sided *P*values for the test of significance of Pearson’s *R*. **f**, UMAP of mosaic PDXs in an ARS-1620 in vivo GENEVA experiment where NSG mice were treated with ARS-1620 (100 mg per kg (body weight)) for 16 days. Cells are colored by G12C status (left) and ARS-1620 or vehicle treatment (right). **g**, Numbers of cells in an apoptotic cell state were counted in ARS-1620 and vehicle treatment samples across *KRAS*^G12*^ mutation status. The *P* value was calculated using a two-sided *χ*^2^ test. Source data

**Extended Data Fig. 2:**
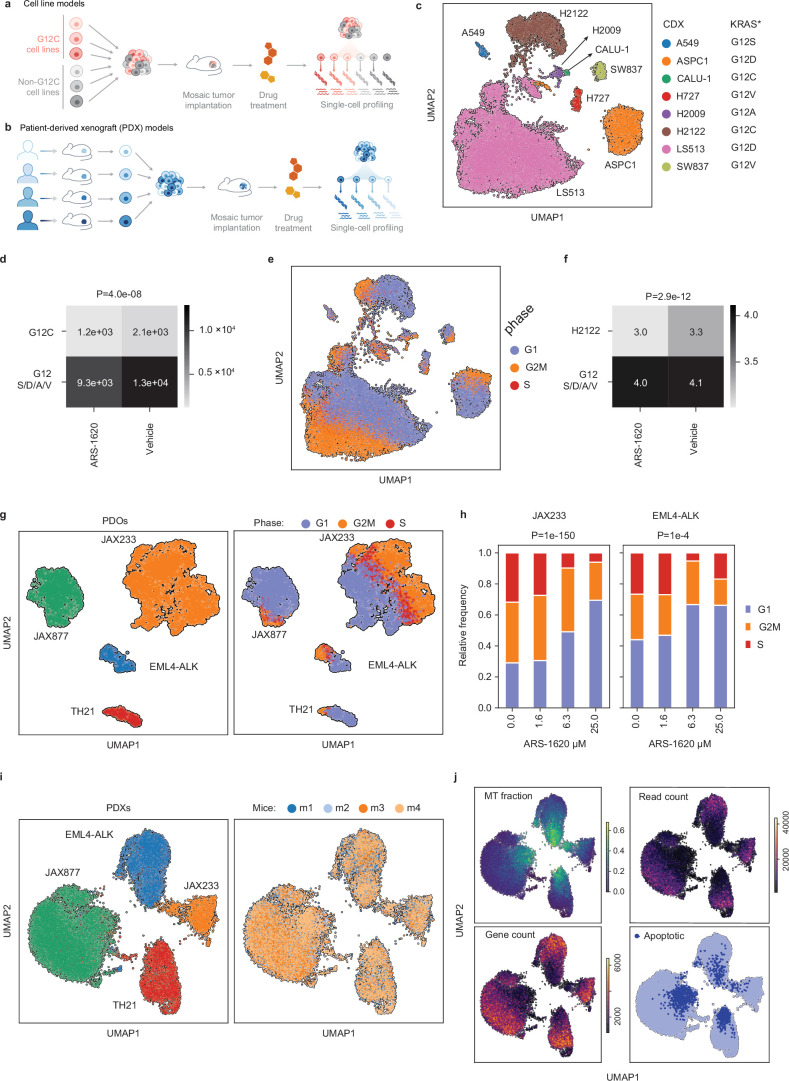
*In vivo* applications of GENEVA. **(a,b)** Schematic of GENEVA for cell line derived xenografts. Mosaic populations are formed by pooling cell lines of interest followed by implantation in mice. Here, we have performed subcutaneous injections, however, for cell lines of similar origin orthotopic injections can also be performed. Once the mosaic tumors have grown, the xenografted mice can be treated with the drugs of interest for several days prior to tumor extraction. Once extracted, the tumors are dissociated and subjected to mouse cell removal and single cell profiling. Two focused experiments are illustrated using **(a)** cell line-derived xenograft models and **(b)** patient-derived xenograft models. **(c)** Cell line-derived xenograft GENEVA pool visualized as UMAP colored by cell line identity. **(d)** Contingency table showing significance of cell line counts in *KRAS* G12C and non-G12C across ARS-1620 and vehicle conditions. Two-sided χ² test was used to calculate P-value, with 2 G12C lines and 6 G12S/D/A/V lines for both ARS-1620 and Vehicle. **(e)** CDX pool UMAP colored by cell cycle phase. **(f)** Contingency table showing significance of log10-cell line counts in H2122 vs non *KRAS* G12C lines across ARS-1620 and vehicle conditions. Two-sided χ² test was used to calculate P, with 6 non-G12C lines for both ARS-1620 and Vehicle. **(g)** UMAP of PDOs grown in GENEVA mosaic colored by PDX identity (left) and cell cycle phase (right) after treatment with multiple doses of ARS-1620. **(h)** G1 arrest of PDXs across increasing ARS-1620 dose shown as proportion of cells in each cell cycle phase. Two-sided χ² test was used to calculate P. **(i)** PDX *in vivo* GENEVA pool visualized as UMAP. Cells are colored by PDX identity and mouse replicates. **(j)** Cells are further colored by several metrics calculated on an individual cell basis: reads from mitochondrial fraction, total read count, total genes detected, and apoptotic cell state. See detailed methods for scoring methods for single-cell transcriptomes. Source data

In vivo oncology xenograft models are particularly limited by the inability to effectively test multiple perturbations across multiple models simultaneously due to the technical variation between xenografted mice that necessitates large numbers of mice to achieve sufficient statistical power. Thus, by endogenously controlling the observation of a pool of multiple cell lines within a single mouse, GENEVA can minimize technical variation in vivo while reproducibly collecting data across genetic backgrounds. We therefore set out to adapt GENEVA for in vivo studies using established patient- and cell line-derived xenograft (PDX and CDX, respectively) models (Extended Data Fig. 2a,b). First, we created flank xenograft mosaic tumors comprising various *KRAS*^G12*^ mutant cell lines across multiple cancer indications (Extended Data Fig. 2a,c). Following injection of the mosaic tumor, we treated mice with either ARS-1620 (100 mg per kg (body weight)) or vehicle for 10 days. After treatment, we resected the tumors, dissociated them into single cells, enriched for cancer cells through a mouse cell removal step and performed scRNA-seq (Fig. 2a and Extended Data Fig. 2c). As expected, we observed significantly fewer cells from the *KRAS*^G12C^-mutant lines after ARS-1620 treatment (Fig. 2b and Extended Data Fig. 2d). Leveraging the transcriptomics, we also used annotated cell cycle genes to examine the effect of ARS-1620 on cell cycle phase across lines (Extended Data Fig. 2e). Consistent with the known cytostatic effect of KRAS inhibitors^19,20^, we found that for certain lines (for example, H2122), KRAS-G12C inhibition led to not only lower cell counts (Extended Data Fig. 2f) but also an increased proportion of cells in G1 (Fig. 2c and Extended Data Fig. 2g,h).

We next validated these findings in patient-derived organoids (PDOs), pooling two *KRAS*^G12C^ models (JAX233 and JAX877) and two non-G12C models (EML4-ALK and TH21) and treating them with increasing concentrations of ARS-1620 or DMSO (Fig. 2d). Consistent with our CDX results, G12C PDO lines showed a dose-dependent increase in the fraction of cells in G1 phase (Fig. 2e). We then tested in vivo GENEVA with these same four models as PDXs in mosaic flank xenografts. Pools were injected into mice, and after tumor formation (48 h), mice were treated with 100 mg per kg (body weight) ARS-1620 or vehicle (two mice per condition) for 16 days. We observed robust representation of all four models within the mosaic tumors (Fig. 2f and Extended Data Fig. 2i) and no significant change in cell counts, as expected for the duration of these experiments. However, analogous to the aforementioned GENEVA platforms, we observed a significant increase in the number of apoptotic cells among G12C PDXs with ARS-1620 treatment (Fig. 2g and Extended Data Fig. 2j).

Together, these GENEVA datasets enable direct assessment of various phenotypic scores across different genetic backgrounds, treatments and doses. In the following sections, we leverage the rich transcriptomic phenotypes and intrapopulation variations frequently seen in our data to highlight how GENEVA can provide insight into molecular mechanisms of drug action and tolerance.

### KRAS-G12C inhibition increases the activity of the mitochondrial electron transport chain

**Fig. 3:**
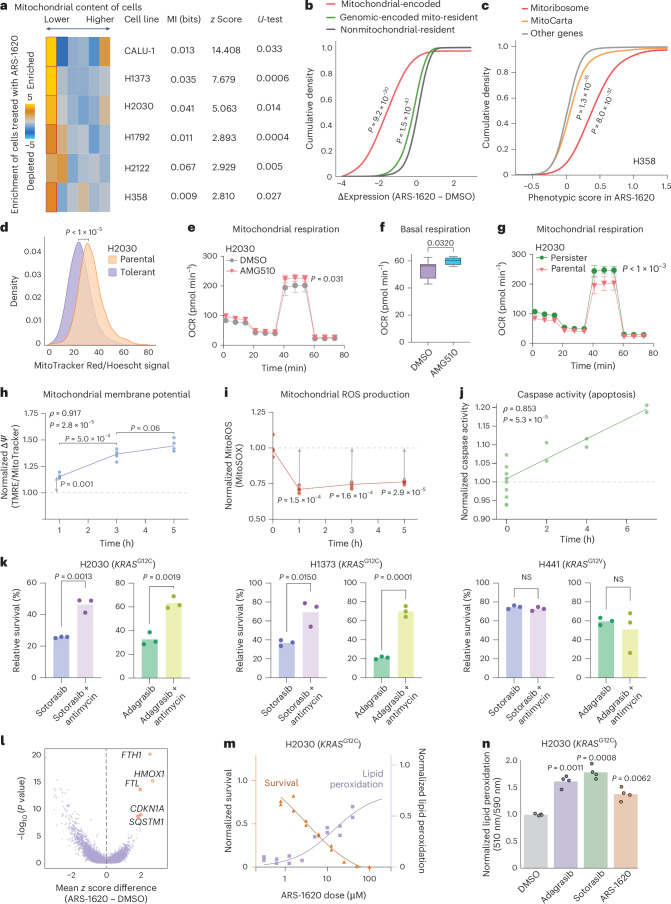
KRAS-G12C inhibition leads to cell death via mitochondrial respiratory activity. **a**, Cells surviving ARS-1620 treatment are enriched among those with lower mitochondrial content. For this analysis, cells were binned by expression of mitochondrial genes from low content (left) to high content (right). For each cell line in the GENEVA pool, the number of cells that were treated with ARS-1620 and reside in each bin was used to calculate an enrichment (gold) or depletion (blue) score. The significance of the pattern was measured using mutual information (MI) and its associated *z* score as well as a two-sided Mann–Whitney *U*-test. **b**, Differential expression following ARS-1620 treatment across mitochondrial-encoded, genomically encoded but mitochondrial-resident (Genomic-encoded mito-resident) and nonmitochondrial-resident protein-coding genesets visualized as cumulative distributions. Lower differential expression indicates downregulation after treatment. Statistics were calculated using a two-sample *t*-test for each geneset using the nonmitochondrial-resident genes as the null distribution. **c**, Genome-wide CRISPRi screening data from the *KRAS*^G12C^-mutant H358 cell line in a synthetic lethal screen with ARS-1620 grouped by MitoCarta, Mitoribosome and all other genes. An increase in phenotypic score indicates a buffering or rescuing effect on cell survival in a drug treatment context. Data are from Lou et al.^21^. **d**, H2030 persistor and parental cell lines profiled for mitochondrial content by MitoTracker Red. Data were compared between parental (*n* = 2,996) and persistor (*n* = 6,415) populations by two-sided Mann–Whitney *U*-test, *P* = 2.75 × 10^−335^. **e**, Oxygen consumption rate (OCR) in H2030 measurements after acute treatment with sotorasib for 2 h at 500 nM using a Seahorse assay. *P* values were calculated by two-sided Welch’s *t*-test (*n* = 10); *n* represents the number of biological replicates in the Seahorse assay, that is, wells with cells treated with drug and measured. Shown is the mean and s.d. **f**, Basal respiration in H2030 cells after acute treatment with sotorasib. Data are from a Seahorse assay. *P* values were calculated by two-tailed *t*-test (*n* = 10); *n* represents the number of biological replicates in the Seahorse assay, that is, wells with cells treated with drug and measured. Shown is the mean and s.e.m. For all box plots, the center lines indicate median values, the box bounds indicate 25th and 75th percentiles, whiskers extend to minimum and maximum values within 1.5× the interquartile range (IQR) from Q1/Q3, and individual points beyond whiskers represent outliers. **g**, OCR measurements after acute treatment with sotorasib for 2 h at 500 nM with a Seahorse assay in parental (green) and persistor (pink) lines. A *P* value of 0.0003 was calculated by two-sided Welch’s *t*-test (*n* = 10 for parental and *n* = 12 for persistor biological replicates). Shown is the mean and s.d.; *n*represents the number of biological replicates in the Seahorse assay, that is, wells with cells treated with drug and measured. **h**, Mitochondrial membrane potential measured by TMRE and MitoTracker Red ratio in flow cytometry assays after induction with sotorasib (500 nM) for 0–5 h. Spearman correlation and associated *P*value are shown calculated across all time points to measure the trend. Individual time point statistics were calculated with Welch’s two-sided *t*-test for 1 h versus 3 h and 3 h versus 5 h. A one-sample two-sided *t*-test was used to calculate statistics for the 1 h against the normalized DMSO values. Data points shown are biological replicates, with each data point representing the median fluorescence across all cells within a single well. **i**, Mitochondrial ROS (MitoROS) formation measured by MitoSOX signal in flow cytometry assays after induction with sotorasib (500 nM) for 0–5 h. A one-sample two-sided *t*-test was used to calculate statistics for the 1-, 3- and 5-h time points against the normalized DMSO values. Data points shown are biological replicates, with each data point representing the median fluorescence across all cells within a single well. **j**, Caspase-3/caspase-7 cleavage measured by CellEvent Caspase-3/7 signal in flow cytometry assays after induction with sotorasib (500 nM) for 0–7 h. Spearman correlation and associated *P*value are shown calculated across all time points to measure the trend. Data points shown are biological replicates, with each data point representing the median fluorescence across all cells within a single well. **k**, Cell survival proportions with sotorasib and adagrasib combination dosing with antimycin A in two *KRAS*^G12C^ cell lines (H2030 and H1373) and one non-G12C cell line (H441). *P* values were calculated by two-tailed *t*-test. Bar plots represent the mean of the data (*n* = 3, biological replicates); NS, not significant. **l**, Volcano plot visualizing differentially expressed genes across ten *KRAS*^G12C^ cell lines in GENEVA pools after treatment with ARS-1620. *P*values were calculated by two-sample *t*-test scoring independent cell lines as biological replicates. **m**, Relative cell survival and lipid peroxidation dose–response to ARS-1620 after 3 days of treatment as measured by CTG and BODIPY 581/591 C11. The lefthand *y*axis indicates survival, subsequently plotted as orange data points, and the righthand *y*axis indicates lipid peroxidation, subsequently plotted as purple data points (*n* = 2 for all doses, biological replicates). **n**, Lipid peroxidation levels, measured by BODIPY 581/591 C11, after treatment with three KRAS-G12C inhibitors compared to vehicle treatment. A two-sample two-sided *t*-test was used to calculate statistics for each treatment condition against the normalized DMSO values. Data points shown are biological replicates, with each data point representing the median fluorescence across all cells within a single well. Source data

**Extended Data Fig. 3:**
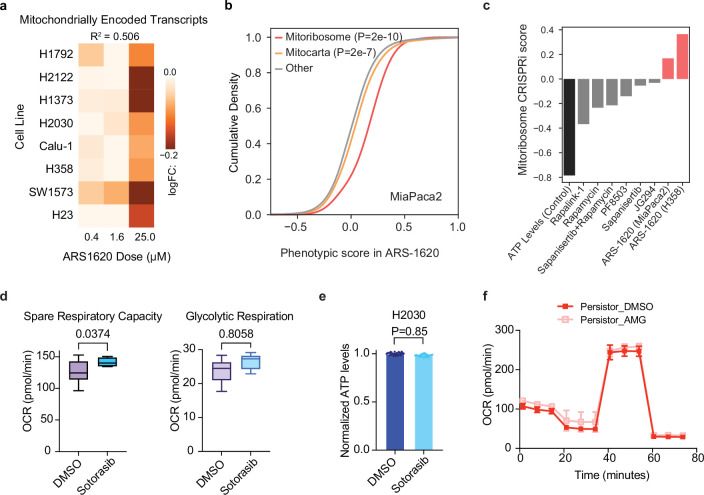
G12C Inhibition and correlation to mitochondrial signatures and changes in activity. **(a)** Mitochondrial transcripts across GENEVA pools at escalating doses of ARS-1620, normalized to vehicle-treated pools. Colors shown reflect Log_2_ fold-change of mitochondrial transcripts for cell lines at different doses relative to vehicle. R^2^ was calculated by treating log_2_FC values as the response variable, cell lines as replicates, and dose as the independent variable. **(b)** Genome-wide screen data from *KRAS* G12C mutant MIA PaCa-2 lines in a synthetic lethal screen with ARS-1620 grouped by MitoCarta, Mitoribosome, and all other genes. Increase in phenotypic score indicates a buffering or rescuing effect on cell survival in a drug treatment context. Genes were treated as replicates within a geneset (Mitoribosome, Mitocarta) and compared using a two sample *t*-test against the majority of other genes in the genome-wide screen data (ie non-Mitoribosomal, non-Mitocarta). **(c)** Median phenotypic score for mitoribosomal genes in multiple genetic screens. **(d)** Spare respiratory and glycolytic respiration in H2030 after acute treatment with Sotorasib as measured by OCR. Sample sizes (biological replicates): spare respiratory capacity, n = 10 (DMSO) and n = 4 (sotorasib); glycolytic respiration, n = 33 (DMSO) and n = 12 (sotorasib). P-values are obtained using Tukey’s test. For all box plots: center lines indicate median values, box bounds indicate 25th and 75th percentiles, whiskers extend to minimum and maximum values within 1.5× IQR from Q1/Q3, and individual points beyond whiskers represent outliers (not shown for glycolytic respiration). **(e)** ATP levels as measured by CellTiter Glo after acute treatment with Sotorasib at 2 h, 500 nM. Sample sizes (biological replicates): n = 30 (DMSO), n = 30 (Sotorasib). P-values were obtained using a two sample *t*-test. **(f)** Persistor population after long-term ARS-1620 treatment is treated with acute Sotorasib and assayed by Seahorse respirometer. Sample size (biological replicates): n = 10 (DMSO) and n = 4 (AMG510/sotorasib) at each timepoint. No significant difference observed by a two sample t-test across all respiratory compartments. Data are presented as mean values +/ standard deviation. Source data

With access to G12C inhibitor-focused GENEVA datasets across various models, we sought to understand the universal mechanisms of resistance to these therapies both in vivo and in vitro. First, we focused on data from our in vitro G12C-focused pool with multiple doses of ARS-1620 (Fig. 1d). We observed that across all *KRAS*^G12C^-mutant lines, cells that survived ARS-1620 treatment had significantly fewer mitochondrial transcripts than their untreated counterparts (Fig. 3a and Extended Data Fig. 3a). To explore this, we performed differential gene expression analysis between treated and untreated cells across all G12C lines. This revealed that transcripts of mitochondrial origin (but not nuclear-encoded, mitochondrial-targeted transcripts) were significantly downregulated after ARS-1620 treatment (Fig. 3b). We also analyzed two published genome-scale ARS-1620 synthetic lethal CRISPR interference (CRISPRi) screens^21^ and observed that in two G12C lines (H358 and MIA PaCa-2), knockdown of mitoribosomal and mitochondria-associated genes significantly increased survival^18,22^ (Fig. 3c and Extended Data Fig. 3b). To determine whether the effect was ARS-1620 specific, we analyzed similar synthetic lethal CRISPRi datasets for other drugs^23–25^. As a positive control, we also included a genetic screen for ATP levels^26^, and, as expected, this dataset showed that silencing mitoribosomal genes resulted in significantly reduced ATP (Extended Data Fig. 3c). Yet, of the datasets we analyzed, none phenocopied the ARS-1620 screens, highlighting the specificity of mitochondrial involvement in ARS-1620 sensitivity (Extended Data Fig. 3c). Together, these results indicate that inhibiting mitochondrial function or reducing mitochondrial content results in increased tolerance to ARS-1620.

To further explore this mitochondrial phenotype, we generated an ARS-1620 persister derivative of the H2030 cell line by treating this *KRAS*^G12C^ line with ARS-1620 for 34 days. Consistent with our GENEVA studies, the H2030 ARS-1620 persister line showed significantly lower mitochondrial content than the drug-naive parental cell line based on MitoTracker-DeepRed staining (Fig. 3d). To test the specificity of this signal to KRAS while also assaying mitochondrial function, we used the clinically approved KRAS-G12C inhibitor sotorasib. With a Seahorse respirometer^27^, we measured oxygen consumption dynamics with and without sotorasib treatment in a 2-h post-treatment window and observed a rapid and significant sotorasib-induced increase in basal respiration (Fig. 3e). This effect is attributable to the increase in spare respiratory capacity at the site of the electron transport chain (ETC), with no significant change in nonmitochondrial-derived respiration or ATP production (Fig. 3f and Extended Data Fig. 3d,e). This sotorasib-induced increase in spare respiratory capacity was absent in H2030 persister cells, highlighting the epistatic interaction between mitochondrial function and KRAS inhibition (Fig. 3g and Extended Data Fig. 3f) and indicating a potential adaptive mechanism in persister cells. Persister cells also displayed higher baseline spare respiratory capacity than parental cells, suggesting that these cells may have already maximally increased mitochondrial activity to balance lower mitochondrial content. This marked increase in mitochondrial respiration was unexpected, as most inhibitors of the ETC lead to decreased mitochondrial respiration^28^. We further characterized this mitochondrial hyperactivity phenotype by conducting an acute time course study measuring mitochondrial membrane potential, mitochondrial reactive oxygen species (ROS) and caspase cleavage in nonpersister cells using flow cytometry sensors. The initial cellular response to KRAS-G12C inhibition was marked by an increase in mitochondrial membrane potential (tetramethylrhodamine, ethyl ester, perchlorate; TMRE) at 1 h after treatment, consistent with the observed acute upregulation in mitochondrial oxygen consumption (Fig. 3h). In tandem, mitochondrial ROS production measured by MitoSOX decreased at 1 h after treatment, whereas caspase-3/caspase-7 cleavage steadily increased 0 to 7 h after treatment (Fig. 3i,j). Although KRAS-G12C inhibition has been shown to induce caspase-3/caspase-7 cleavage, our data show that strong mitochondrial hyperactivity precedes caspase cleavage and traditional mechanisms of cell death.

Given the impact of KRAS-G12C inhibition on elevated mitochondrial activity in the acute phase of drug response, we hypothesized that sotorasib may act via the ETC to induce cell death. To investigate this, we used antimycin A1, a complex III inhibitor, to determine if selective inhibition of the rate of electron flux through the ETC could rescue lethality of sotorasib in G12C cell lines. We combined antimycin A1 treatment with either sotorasib or adagrasib in CellTiter-Glo (CTG) survival assays and found that antimycin A1 attenuates lethality of both KRAS-G12C inhibitors in G12C lines (H2030 and H1373) but not in a G12V line (H441; Fig. 3k). Together, the data demonstrate that KRAS-G12C inhibitors induce cell death via a mechanism of increased mitochondrial activity that is specific to *KRAS*^G12C^ mutants, indicating an on-target effect of KRAS-G12C inhibitors.

**Extended Data Fig. 4:**
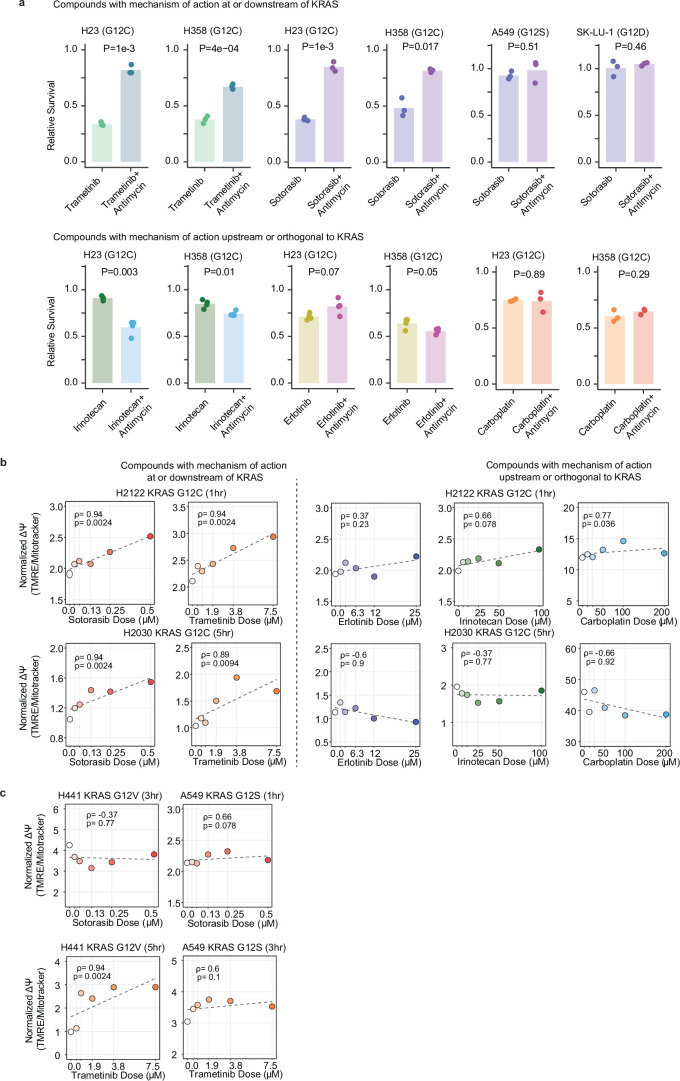
G12C inhibition and non-G12C inhibition effects on antimycin-cotreated cell survival and mitochondrial membrane potential. **(a)** Cell survival proportions with antimycin combination dosing with drugs targeting different pathways in *KRAS* G12C cell lines as measured by 72 hour Cell Titer Glo assay. Sample size (biological replicates): n = 3 for each group. No rescue was observed with addition of antimycin in pathways upstream or orthogonal to hyperactive KRAS. **(b)** Mitochondrial membrane potential measured by TMRE and MitoTracker Red ratio in flow cytometry assays after dose-dependent acute treatment with drugs targeting different pathways in *KRAS* G12C cell lines. Sample size (biological replicates): n = 3 for each concentration group. The points shown are the median for each concentration, and the Spearman correlation and associated p-values are reported as calculated across multiple concentrations. **(c)** Mitochondrial membrane potential measured by TMRE and MitoTracker Red ratio in flow cytometry assays after dose-dependent acute treatment with drugs targeting different pathways in *KRAS* G12V/S cell lines. Sample size (biological replicates): n = 3 for each concentration group. The points shown are the median for each concentration, and the Spearman correlation and associated p-values are reported as calculated across multiple concentrations. Source data

To investigate whether this observed mitochondrial activation is specific to KRAS-G12C inhibitors rather than a nonspecific effect of anticancer agents (Extended Data Fig. 4a–c), we compared other MAPK pathway inhibitors (trametinib and erlotinib) and orthogonal agents (irinotecan and carboplatin) to sotorasib. Of this drug panel, only inhibitors targeting KRAS-G12C (sotorasib) or downstream signaling (trametinib) induced mitochondrial hyperactivation that could be rescued by antimycin A1. This mitochondrial hyperactivity response pattern across drugs was further validated in G12C lines (H2122 and H2030) using TMRE and MitoTracker staining. Again, sotorasib and trametinib induced a dose-dependent increase in mitochondrial respiration, whereas erlotinib, irinotecan and carboplatin did not. Furthermore, for a G12V line, sotorasib failed to induce mitochondrial activity, but trametinib did, confirming the specificity of the mechanism to KRAS-G12C signaling. Overall, this supports a working model that mitochondrial hyperactivation is an effect specific to KRAS-G12C and downstream MAPK inhibition rather than a general effect of cell death.

**Extended Data Fig. 5:**
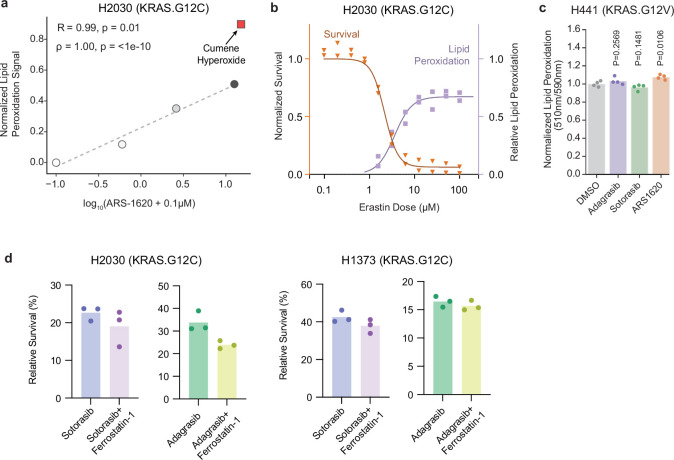
Effects of G12C inhibition on lipid peroxidation and ferroptosis pathways. **(a)** Lipid Peroxidation measurement of cells by flow cytometry after dose titration of ARS-1620. Lipid peroxidation measurements were performed at 24 h post-treatment using BODIPY C11 reagent and compared to Cumene Hyperoxide, a known rapid inducer of lipid peroxidation as a control. Sample size (biological replicates): n = 3 for each concentration group. The points shown are the median for each concentration, and the Spearman correlation and associated p-values are reported as calculated across multiple concentrations. **(b)** Relative cell survival and lipid peroxidation levels dose-response to Erastin. Survival was measured using CTG at 72 hours and lipid peroxidation with BODIPY C11 at 24 hours. Left axis shows normalized survival and the right axis shows normalized lipid peroxidation. **(c)** Lipid peroxidation values after treatment with three *KRAS* G12C inhibitors compared to a vehicle in H441 (*KRAS* G12V). P-values are obtained using a two-tailed Student’s t-test against vehicle (DMSO). Sample size (biological replicates): n = 4 for each group. **(d)** Cell survival proportions with Sotorasib and Adagrasib combination dosing with Ferrostatin-1 in two *KRAS* G12C cell lines (H2030 and H1373) and one non-G12C cell line (H441). Sample size (biological replicates): n = 3 for each group. No rescue was observed with addition of Ferrostatin-1 pretreatment for 24 hours. Source data

A secondary signal from our focused KRAS-G12C GENEVA pool suggested that KRAS-G12C inhibitors could also be inducing ferroptosis. Across G12C lines surviving drug treatment, we found an upregulation of antiferroptotic genes, including two components of the ferritin complex *FTH1* and *FTL* (Fig. 3l). We further investigated this signal by measuring lipid peroxidation induced by ARS-1620 using the flow cytometry live-cell lipid peroxidation sensor BODIPY C11. We observed that ARS-1620 induced lipid peroxidation in a dose-dependent manner (Extended Data Fig. 5a) and that cell survival decreased as the amount of lipid peroxidation increased (Fig. 3m). A comparison of the dynamics of these two phenotypes, survival and lipid peroxidation, between ARS-1620 and the known ferroptosis-inducing erastin showed similar dose-dependent relationships of decreased survival and increased lipid peroxidation (Extended Data Fig. 5b). To characterize the generalizability of this phenotype to multiple KRAS-G12C inhibitors, we performed similar lipid peroxidation measurements^29^ in response to adagrasib, sotorasib and ARS-1620 in G12C and non-G12C cell lines. We found that all three compounds significantly increased lipid peroxidation in a G12C line (H2030; Fig. 3n), whereas there was no significant change in a non-G12C line (H441; Extended Data Fig. 5c). However, unlike antimycin A1, in a rescue experiment with co-treatment of KRAS-G12C inhibitors and the antiferroptotic agent ferrostatin-1 (ref. ^30^), we did not observe decreased lethality to KRAS-G12C inhibitors, indicating that although KRAS-G12C inhibitors induce ferroptotic markers, this is not a primary mechanism of action for lethality (Extended Data Fig. 5d). In conjunction, these examples of mitochondrial activation and ferroptosis illustrate how GENEVA can uncover primary and secondary mechanisms of drug action.

### Discovering and targeting the mechanisms of tolerance to KRAS-G12C inhibition in vivo and in vitro

**Fig. 4:**
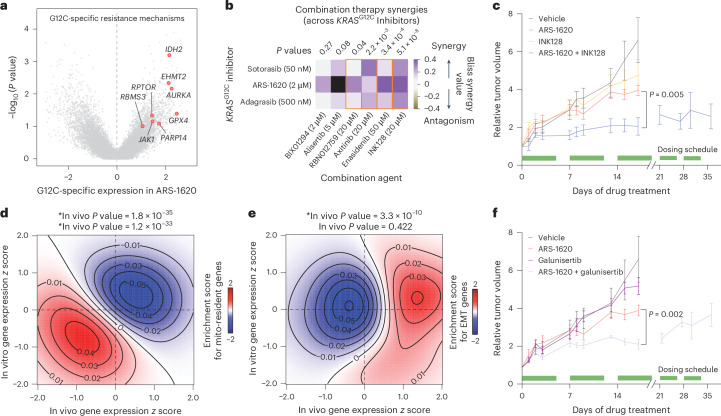
Targeting pathways associated with increased tolerance to KRAS-G12C inhibition leads to more efficacious combination therapies. **a**, Volcano plot of *KRAS*^G12C^-mutant cell lines showing *KRAS*^G12C^-specific gene expression upregulation. G12C-specific expression was taken by first calculating differential expression between DMSO and ARS-1620 treatment for each cell line and then taking the mean log_2_ (fold change) difference between cell lines with G12C (*n* = 8) and non-G12C (*n* = 2) mutations. *P*values were calculated using two-tailed *t*-tests between G12C and non-G12C groups. Druggable proteins significantly upregulated in a G12C-specific manner are highlighted in red. **b**, Combination therapy testing with three KRAS-G12C inhibitors and compounds targeting G12C-specific resistance mechanisms identified in **a** was performed in a 3-day CTG survival assay in H2030 cells. Data are shown with Bliss synergy values colored as a heat map, with purple indicating Bliss drug synergy, gray indicating Bliss antagonism and an orange outline indicating *P* values less than 0.05. *P*values were calculated by two-tailed *t*-test of biological replicates specific to a dual-agent combination compared against a null distribution estimated from no drug biological replicates (*n* = 4). **c**, Combination therapy in vivo study performed in NSG mice with treatment duration between 17 and 35 days. ARS-1620 (100 mg per kg (body weight)) was coadministered with the mTOR inhibitor INK128 (0.3 mg per kg (body weight)). ARS-1620 and INK128 were dosed singly and in combination daily during the indicated dosing schedule intervals (green) by intraperitoneal injection with *n* = 5 NSG mice per group. The data are shown as mean ± s.d. Statistical significance of differences in final normalized tumor volumes between INK128 and ARS-1620 + INK128 treatment groups was determined by two-sided *t*-test. **d**, Two-dimensional density plot showing concordance of gene expression downregulation between in vivo and in vitro mosaic pools. Red indicates mitochondrially encoded gene expression enrichment, and blue indicates depletion. Expression data were transformed to differential expression *z* scores by comparison of ARS-1620 to DMSO conditions for respective in vitro and in vivo GENEVA pools. *P* values were calculated by two-sided Mann–Whitney *U*-test of *z* scores from genes within the geneset shown and all other genes. *P*values indicate the significance of downregulation in each model system (in vitro/in vivo). **e**, Two-dimensional density plot showing model-specific downregulation of gene expression between in vivo and in vitro mosaic pools. Red indicates EMT geneset enrichment, and blue indicates depletion. Statistics were calculated as described in **d**. **f**, Combination therapy in vivo study with TGFβ/EMT inhibitor galunisertib (75 mg per kg (body weight)) and ARS-1620 (100 mg per kg (body weight)). ARS-1620 and galunisertib were dosed singly and in combination daily during the indicated dosing schedule intervals (green) by intraperitoneal injection with *n* = 5 NSG mice per group. Data are shown as mean ± s.d. Statistical significance of differences in final normalized tumor volumes between galunisertib and ARS-1620 + galunisertib treatment groups was determined by two-sided *t*-test. Control and ARS-1620 mouse data are shared between **c** and **f**. Study design and statistics were as described in **c**. Source data

**Extended Data Fig. 6:**
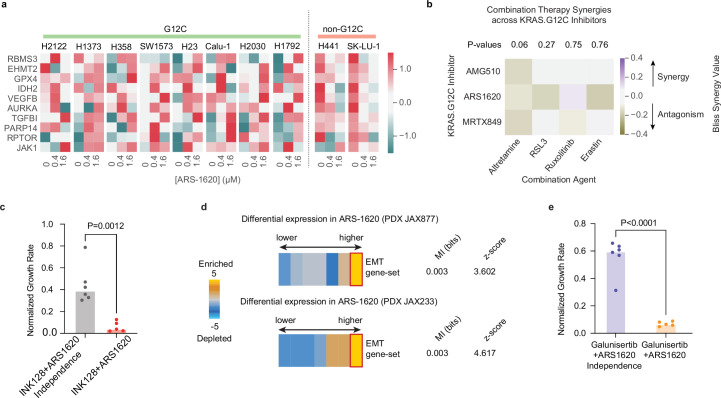
Discovery of mechanisms of resistance to *KRAS* G12C inhibition. **(a)** Heatmap of gene expression of various G12C specific mechanisms across individual cell lines and ARS-1620 dose. **(b)** Bliss synergy of combination of *KRAS* G12C inhibitors and three ferroptosis inducers and Ruxolitinib. *P-values* were calculated by two-tailed t-test of replicates specific to a dual-agent combination compared against a null distribution estimated single agent activities. **(c)** *In vivo* combination studies of INK128 + ARS-1620 visualized as the expected additive effect on growth rate of tumors and the observed *in vivo* growth rate. Sample size (biological replicates): n = 6 (independence), n = 5 (empirical). P-value obtained using a two-sample *t*-test. **(d)** EMT upregulation in PDX GENEVA pools for two *KRAS* G12C mutant PDX after longitudinal drug treatment. The EMT gene-set is significantly enriched in the top-most gene expression bin for both JAX877 and JAX233 PDX models. Shown are the enrichment (gold) and depletion (blue) patterns of this gene-set across the differential expression bins, as well as their association mutual information (MI) and *z*-score. **(e)** *In vivo* combination studies of INK128 + ARS-1620 visualized the expected additive effect on growth rate of tumors and the observed *in vivo* growth rate. Sample size (biological replicates): n = 6 (independence), n = 5 (empirical). P-value is obtained using a two-tailed Student’s t-test, p = 1.23 × 10^−5^. Source data

As shown in Extended Data Fig. 1d, there is apparent heterogeneity in drug sensitivity within *KRAS*^G12C^ models, and comparing these surviving subpopulations across sensitive lines could reveal general mechanisms of resistance to KRAS-G12C inhibition. We therefore performed a differential expression analysis focused on genes differentially expressed in ARS-1620-treated cells in a KRAS-G12C-dependent manner (Fig. 4a and Extended Data Fig. 6a). We observed higher expression of several druggable protein-coding genes as candidates for conferring resistance in *KRAS*^G12C^-mutant cells, including *JAK1*, *RPTOR*, *PARP14*, *GPX4*, *AURKA*, *EHMT2* and *IDH2*. Several of these pathways, such as mTOR and AURKA, have already been implicated in drug resistance. To test these candidates, we used the following panel of compounds targeting their respective pathways to assess potential synergies with KRAS-G12C inhibition^31,32^: ruxolitinib (JAK), INK128 (mTOR), RBN012759 (PARP), RSL3/erastin/altretamine (ferroptosis), alisertib (AURKA), BIX01294 (histone methyltransferases) and enasidenib (IDH2). For RBN012759, axitinib, enasidenib and INK128, we observed a significant synergy (measured by Bliss scores)^33^ across three KRAS-G12C inhibitors (Fig. 4b). By contrast, consistent with our experiments with ferrostatin-1, ferroptosis inducers did not synergistically interact with KRAS inhibition (Extended Data Fig. 6b). For INK128, which showed the strongest synergy, we also performed in vivo combination therapy with subcutaneous H2030 tumors in xenografted mice. After tumor formation (21 days after injection), the mice were randomly divided into four arms and treated with ARS-1620 (100 mg per kg (body weight)) and INK128 (0.3 mg per kg (body weight)), both as monotherapies and in combination. As expected, the individual drugs reduced tumor growth, while combining them significantly increased efficacy (Fig. 4c and Extended Data Fig. 6c). Thus, GENEVA uncovers cancer cell heterogeneity that can be harnessed to identify generalizable mechanisms of drug resistance and design rational combination therapies for improving the efficacy of targeted therapeutics.

Because GENEVA enables rich profiling of drug response both in vivo and in vitro, we wanted to explore how these two modalities generate different insights into mechanisms of drug action and resistance. To tackle this, we compared *KRAS*^G12C^-specific gene expression changes in response to ARS-1620 both in vivo and in vitro. We observed an overall agreement between these two modalities. For example, mitochondria-resident protein-coding genes, significantly downregulated in ARS-1620-tolerant cells (Fig. 3b), showed a similar pattern both in vivo and in vitro (Fig. 4d). However, a notable difference we observed is that epithelial-to-mesenchymal transition (EMT)-associated genes were significantly enriched among genes upregulated in ARS-1620-tolerant cells in vivo but not in vitro (Fig. 4e). This indicates that cells undergoing EMT may be more resistant to KRAS-G12C inhibition in vivo, an effect not as strongly and universally observed in vitro. Consistently, we noted similar upregulation of EMT gene expression in the two ARS-1620-treated *KRAS*^G12C^ PDX models (Fig. 2f and Extended Data Fig. 6d). This phenotypic divergence between the two modalities may result from the entanglement between EMT and the tumor microenvironment, an in vivo-specific modulator^34^. To test the potential role of EMT in response to KRAS-G12C inhibition, we used ARS-1620 + galunisertib (a transforming growth factor-β (TGFβ) receptor inhibitor^35^) combination therapy to simultaneously target the EMT and KRAS pathways. We observed a significant and synergistic reduction in tumor growth with this combination therapy in xenografted mice treated with 75 mg per kg (body weight) galunisertib (Fig. 4f and Extended Data Fig. 6e). Together, our results demonstrate that EMT plays a role in in vivo resistance to KRAS-G12C inhibition, and in vivo GENEVA reveals additional pathways that may go unnoticed in vitro.

**Extended Data Fig. 7:**
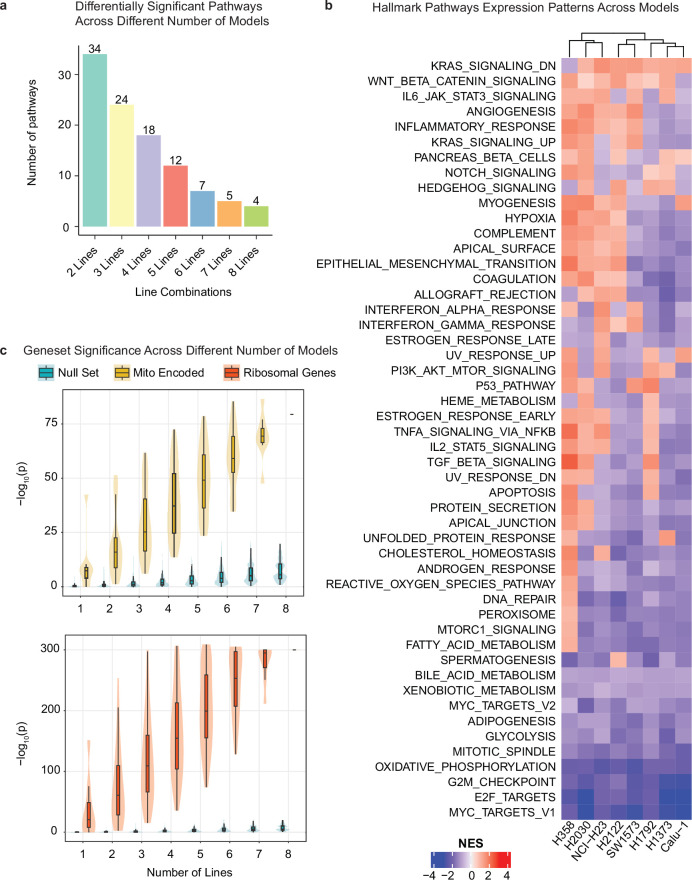
Benchmarks for the impact of model size on GENEVA’s analytical performance. **(a)** Downsampling of multiple cell lines from GENEVA data and significant geneset number as a function of the number of models included. Hallmark pathways were identified in individual cell line comparisons using a significance threshold of padj < 0.05 computed using GSEA. Intersections of these pathways were taken for all combinations of cell lines and the set size of the union of these intersections was plotted. **(b)** Normalized Enrichment Scores (NES) for GENEVA cell lines after *KRAS* G12C inhibitor treatment. NES scores were calculated by geneset enrichment analysis (GSEA) on z-score normalized differential expression values for each cell line, individually. The heatmap shows NES for significantly enriched Hallmark pathways (padj < 0.05) in at least one cell line **(c)** Downsampling of multiple cell lines from GENEVA and individual geneset examination for Mitochondria resident genes and Ribosomal genes across two-sided P-values. P-values for mitochondrial resident and ribosomal genesets were calculated in all combinations of cell lines from GENEVA by combining individual P-values using Fisher’s method. This was compared to P-values calculated in the same way from 100 iterations of randomly sampled null genesets of 100 genes. For all box plots: center lines indicate median values, box bounds indicate 25th and 75th percentiles, whiskers extend to minimum and maximum values within 1.5× IQR from Q1/Q3, and individual points beyond whiskers represent outliers. Source data

Similarly, we further examined the value of studying multiple models and using different biological models as biological replicates in statistical tests for geneset and pathway discovery. We started by downsampling the number of *KRAS*^G12C^ models in our GENEVA pool from eight cell lines to two, and as we decreased the number of lines sampled as biological replicates, we observed a concomitant decrease in the number of overlapping significant gene sets. Therefore, including more models in this framework enables detection of shared significant signals despite underlying differences in gene expression changes (Extended Data Fig. 7a,b). To demonstrate the utility of including multiple models in discovering significant gene sets more quickly, we performed a similar analysis but calculated the change in significance as measured by the *P*value of each gene within gene sets based on differential expression scoring. We found that for the two gene sets that led us to the mitochondrial and mTOR hypotheses (mitochonria-encoded and ribosomal genes), the significance increased as the size of the biological model set increased (Extended Data Fig. 7c). Conversely, the null dataset taken as a random sampling of gene sets did not increase dramatically in significance (Extended Data Fig. 7c).

### Validation of mechanisms of tolerance and resistance to KRAS-G12C inhibition, prioritized from GENEVA, using in vivo genetic screens

**Fig. 5:**
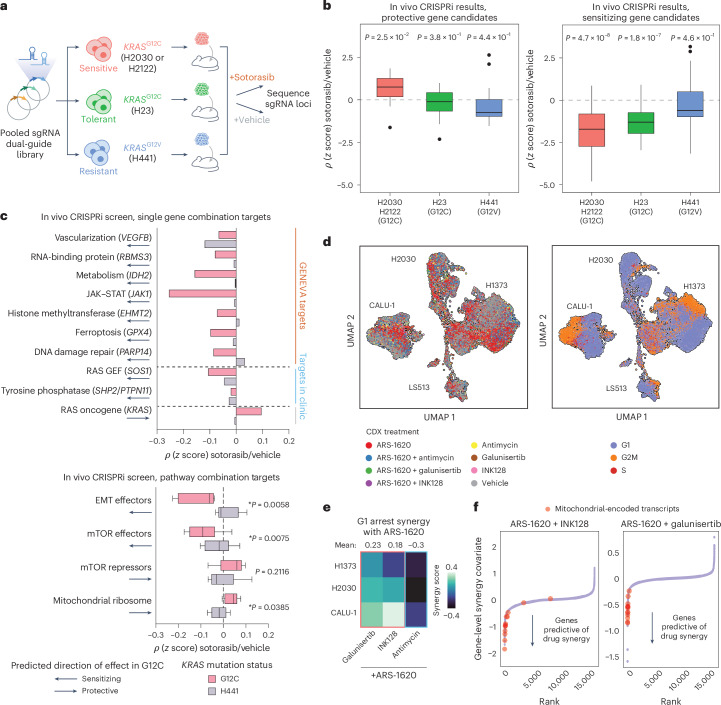
GENEVA enables multiplexed combination therapy screening in vivo*.* **a**, Schematic illustrating pooled genetic in vivo screens in multiple *KRAS*^G12*^ cancer models. **b**, In vivo CRISPRi screen results illustrate differences between cancer models for protective and sensitizing gene candidates in aggregate (*n* = 46 genes for each group). Protective and sensitizing gene candidates were annotated based on GENEVA data and known pathway direction of effect; *ρ* scores were computed for each condition against the paired vehicle screen, and statistics were computed with a one-sample *t*-test against a null *ρ* distribution at 0. For all box plots, the center lines indicate median values, the box bounds indicate 25th and 75th percentiles, whiskers extend to minimum and maximum values within 1.5× IQR from Q1/Q3, and individual points beyond whiskers represent outliers. **c**, In vivo CRISPRi screen results broken out by individual driver genes and pathways with GENEVA predictions annotated as arrows (*n* = 46 genes for each group). For comparison, in the individual driver gene breakout plot, known combination targets currently being studied in clinical trials (*SOS1* and *SHP2*) are shown as well as the known binding target (*KRAS*). Statistics were computed for genesets with a two-sample *t*-test for all gene *ρ* values in either the G12C (H2030 and H2122 cells) or H441 cell model. For all box plots, the center lines indicate median values, the box bounds indicate 25th and 75th percentiles, whiskers extend to minimum and maximum values within 1.5× IQR from Q1/Q3, and individual points beyond whiskers represent outliers. **d**, Cell lines xenografted as mosaic tumors were treated with drugs in single or combination dosing (left). Mosaic tumors were grown for 10 days before daily treatment with drug combinations for seven consecutive days by intraperitoneal injection. ARS-1620 (100 mg per kg (body weight)), galunisertib (75 mg per kg (body weight)), INK128 (0.3 mg per kg (body weight)) and antimycin A1 (0.5 mg per kg (body weight)) were administered singly or in combination at these concentrations. Clusters are labeled by the cell line of origin and colored by the drug treatment condition. Cells are colored by their cell cycle state and identity (right). **e**, Bliss synergy values calculated on G1 arrest scores for *KRAS*^G12C^ cell lines from GENEVA after combination dosing with ARS-1620 and galunisertib, INK128 or antimycin. Heat map colors reflect the relative level of synergy for the two agents, with lighter colors indicating synergistic suppression of G1 and darker colors indicating antagonistic effects on G1. **f**, Identification of genes that deviate from additive synergy models in terms of expression change in response to drug treatment. Data from single-agent and dual-agent GENEVA studies were used to build linear models for calculation of gene-level coefficients. The difference between single-agent and dual-agent model coefficients for each gene were calculated and rank ordered; *z* scores of the value of the coefficient difference are plotted on the *y*axis, and the rank of the genes from lowest to highest is plotted on the *x*axis. This analysis was performed for both ARS-1620 + INK128 and ARS-1620 + galunisertib. Mitochondrial genes are colored in red dots on both plots. Source data

To better understand these prioritized combination therapies from GENEVA, we performed pooled in vivo genetic screens in xenograft tumors to knock down genes from several of these pathways using dual-guide dCas9-KRAB inactivation in multiple cell lines with different *KRAS*^G12*^ statuses: (1) H2030 and H2122 cells (G12C lines sensitive to sotorasib), (2) H23 cells (a G12C line known to be moderately tolerant of sotorasib) and (3) H441 cells (a G12V line resistant to sotorasib)^7,36^. Genes included in the knockdown screen were chosen from pathways we previously showcased (mTOR, EMT and mitochondrial ribosomal components), as well as specific genes including *RBMS3*, *EHMT2*, *GPX4*, *IDH2*, *VEGFB*, *PAP14*, *JAK1*, *KRAS*, *PTPN11*(*SHP2*) and *SOS1*. After knockdown and implantation, mice were treated with either sotorasib or vehicle for 13 days before collection and sequencing of single guide RNA (sgRNA) loci to measure guide representation (Fig. 5a).

Based on our GENEVA studies, we categorized each gene as either protective or sensitizing to KRAS-G12C inhibitor (sotorasib) treatment compared to vehicle and observed that, on average, protective candidates had significantly increased *ρ* values (combined guide *z* scores for each gene as a measure of impact on sensitivity) in the sensitive G12C lines (H2030 and H2122), whereas the G12V line (H44) and moderately sensitive G12C line (H23) showed no significant difference (Fig. 5b). This indicates that GENEVA accurately prioritized genes and pathways that would be protective against KRAS-G12C inhibitor treatment. When we examined sensitizing candidates, we found that in all three G12C lines, these genes had significant negative *ρ* values, indicating relative depletion in sotorasib treatment compared to vehicle. This effect was more pronounced in the more sensitive G12C lines (H2030 and H2122), whereas no significant effect was observed in the G12V line (H441).

**Extended Data Fig. 8:**
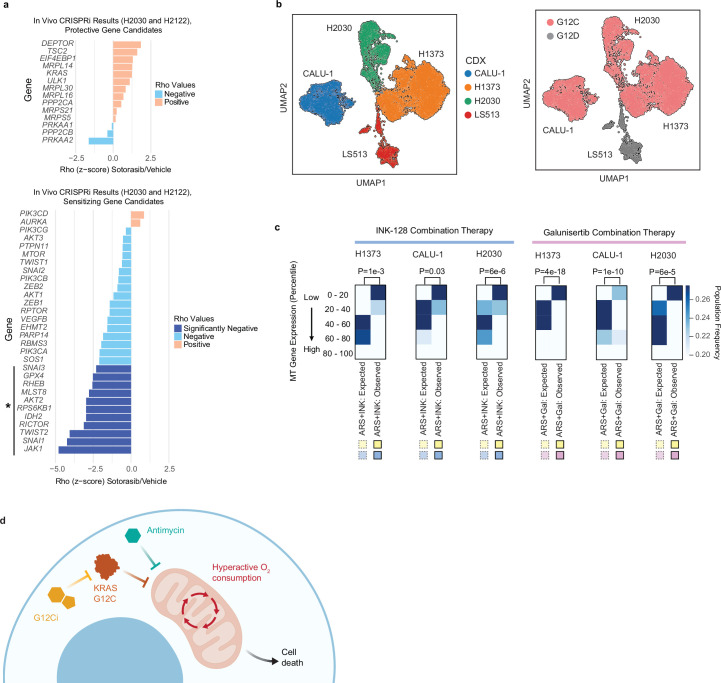
GENEVA enables gene level profiling of *in vivo* drug combinations. **(a)** Individual gene CRISPRi Rho scores for all protective and sensitizing gene candidates as annotated by GENEVA in G12C models H2030 and H2122. Statistics for significant Rho values were computed using as described in the methods. **(b)** UMAP visualization of an *in vivo* GENEVA pool for combination therapy studies colored by cell line identity (left) and G12 mutant allele (right). **(c)** INK128 and Galunisertib with ARS-1620 gene level synergy model. Blue squares indicate enrichment of cells across bins of mitochondrial expression. Gene level synergistic decrease in mitochondrial genes was measured by two tailed t-test. **(d)** Model of *KRAS* G12C pharmacological inhibition’s effect on mitochondrial oxygen turnover leading to cell death. Source data

When analyzing GENEVA-prioritized hits, multiple single-gene targets from different pathways showed significant sensitizing effects in G12C lines (H2030, H2122 and H23) compared to the G12V line (H441; Fig. 5c and Extended Data Fig. 8a). Furthermore, most genes showed greater sensitizing effects than two targets currently studied in clinical trials with KRAS inhibitors (SOS1 and SHP2). Our earlier findings that focused on EMT and mTOR inhibition as key combination pathways and mitochondrial ribosomal components as key rescue pathways for KRAS-G12C inhibitors were also validated in these in vivo screening data. Silencing EMT and mTOR effector genes resulted in significant sensitization to KRAS-G12C inhibitors specific to *KRAS*^G12C^ lines. By contrast, knocking down mitochondrial ribosomal components elicited a significant protective effect.

### Leveraging GENEVA for rich profiling of combination therapies

As shown above, scRNA-seq provides rich phenotyping that far exceeds traditional cell count-based measures of drug sensitivity. The ability of GENEVA, especially in its in vivo modality, to successfully scale this type of molecular phenotyping prompted us to expand our approach beyond monotherapies. To test whether GENEVA could be similarly leveraged to study in vivo drug synergies, we created mosaic tumors composed of three *KRAS*^G12C^ lines (CALU-1, H2030 and H1373) and a G12D line (LS513; Extended Data Fig. 8b). We treated mice harboring mosaic tumors with antimycin A1, galunisertib and INK128, both individually and in combination with ARS-1620. We then used in vivo GENEVA to create a large-scale map of phenotypic and molecular responses to these combination therapies (Fig. 5d) and analyzed the resulting dataset to study drug synergies at the molecular level. For example, we used our scRNA-seq data to measure Bliss drug synergies for causing G1 arrest. Consistent with our previous observations, we observed a positive Bliss synergy between ARS-1620 + galunisertib and ARS-1620 + INK128 across *KRAS*^G12C^ lines (Fig. 5e). By contrast and as expected, antimycin A1 showed a directionally opposite synergy score in *KRAS*^G12C^ lines, indicating rescue of G1 arrest when used in combination with ARS-1620.

To identify the genes and pathways that contribute to these synergies, we used our scRNA-seq data to fit linear models of gene expression based on the cell lines and drug treatment to obtain gene-level coefficients. Just as we previously modeled drug synergy on phenotype by comparing single-agent additive effects to dual-agent observed effects, we sought to find genes that fell outside of simple additive models. We fit a baseline model taken from data on single-agent treatments in GENEVA pools and fit a second model taken from data on single-agent and dual-agent treatments. For each gene, we then calculated gene-level coefficients from the single-agent model and compared these values to coefficients from the dual-agent model. To identify genes that fall outside of an additive model, we ranked genes according to their difference between single-agent and dual-agent models. Consistent with our earlier observations of decreased mitochondrial activity as a resistance mechanism, here, too, mitochondrial genes were negatively downregulated in the dual-agent experimental conditions, ARS-1620 + galunisertib and ARS-1620 + INK128 (Fig. 5f and Extended Data Fig. 8c). Overall, GENEVA enables both multiplexed phenotypic profiling of drug combinations in vivo and gene-level understanding of which genes drive drug synergy.

## Discussion

In this study, we introduced GENEVA and how it reveals the molecular basis of inter- and intrapopulation heterogeneity in drug response by leveraging high-resolution measurements of natural phenotypic variations across a large panel of cancer cell lines and PDXs.

Using GENEVA, we showed that perturbation of mitochondrial respiration is a previously unappreciated mechanism of action of KRAS-G12C inhibitors (Extended Data Fig. 8d). Studies examining the dynamics of KRAS-G12C inhibition using small-molecule therapies have focused on upstream RTK reactivation or downstream rebound mechanisms^37–40^. However, the discovery that mitochondrial content and respiration play a direct role in the persistence of *KRAS*^G12C^-mutant cells under treatment was unexpected perhaps due to prior studies of resistance mechanisms focusing on compensatory pathway reactivation. We also identified pathways of drug resistance (for example, mTOR and EMT) that informed new combination therapies, two of which we validated in vivo using combination studies with ARS-1620. mTOR inhibition has been proposed as a key synergistic avenue for increasing response in individuals treated with KRAS-G12C inhibitors, and our GENEVA studies align with several pharmaceutical companies pursuing this combination in clinical trials.

Second, cancer cells in vivo better capture the tumor microenvironment context in humans and lead to insights more likely to generalize to the clinic^41,42^, which motivated extending pooled single-cell assays to in vivo cancer models. As we showed with the activation of EMT in response to KRAS-G12C inhibition in vivo, there are key genes and pathways that emerge only when studied in vivo. Recent data collected from individuals again support our findings regarding EMT for combination therapy. For example, in a study presenting different types of genetic or nongenetic alterations in individuals refractory to adagrasib, several participants displayed cellular transformation in refractory tumor biopsies accompanied by a lack of any novel genetic alterations^43^. However, histology of these refractory tumors demonstrated a decrease in the number of tight and discrete epithelial foci, which is a hallmark phenotype of EMT. Studies using a combination of refractory cells derived from in vitro models and clinical data also point to a change in cell morphology after resistance to KRAS-G12C inhibitors^44,45^. Together with our finding that EMT is upregulated in *KRAS*^G12C^-mutant cells surviving targeted therapy, there is an emerging hypothesis that cellular transformation is an important aspect of adaptation to KRAS-G12C inhibition. We propose that EMT could be a driver of this resistance and demonstrated that EMT inhibition synergistically improves outcomes in vivo. Furthermore, for EMT, mTOR, mitochondrial ribosomal proteins and other single-gene targets prioritized from GENEVA, we validated the direction of effect in genetic in vivo knockdown experiments. Therefore, GENEVA effectively prioritizes targets and pathways for understanding drug mechanisms of action that robustly validate in orthogonal experiments.

While other methods take advantage of multiplexed scRNA-seq across models, drugs and genetic manipulations, we have extended multiplexed scRNA-seq to a scalable in vivo platform that can also use challenging human-derived models^41,46–48^. For example, PRISM aimed to scalably phenotype many cancer models after drug treatment, but because this technology focused on measuring cell survival after several days in pooled multiplexed models in 2D in vitro culture, it lacked a deeper readout that informed mechanisms of drug action and provided a poor readout of complex cellular responses. More recent work by McFarland et al. with the MIX-seq method demonstrated the application of combining pooled assays with scRNA-seq readouts, building on PRISM-style 2D in vitro assays. However, although MIX-seq provided scRNA-seq readouts, the duration of drug treatment response was limited to 24 h, which limits the observation window and concurrent measurement of phenotypic response. Phenotyping in the context of cancer requires a minimum of several days in vitro but even more in vivo to measure cancer cell death and survival rather than acute transcriptomic responses to treatments. Multiplexed tumor models, therefore, require creation of balanced pools of different cell lines that consider growth dynamics of individual members of the pool and are robust across days to weeks. This process of characterizing lines and balancing the pool is essential for the duration of treatments relevant to the pharmacogenomic measurements of drug sensitivity, tolerance and resistance.

An important limitation of in vivo GENEVA is that, in its present iteration, the mosaic tumors are grown in immunocompromised mice. Therefore, non-cell-autonomous mechanisms of drug response that involve components of adaptive immunity fall are not currently captured. Nevertheless, the vast majority of targeted therapies remain in the cell-autonomous sphere with *KRAS*^G12C^-mutant tumors being a notable example. We also have not tested the maximum number of cell types that can be mixed together in a pooled assay, and there is the possibility that cancer cells from different backgrounds may influence one another. In our studies, cancer cells in the pool grow and respond similarly to their behavior in isolation, including growth rates in monocultures compared to those in GENEVA pools. Furthermore, as the diversity of cells within the pool grows, the likelihood of a specific pair of cells coming into contact diminishes rapidly.

Overall, GENEVA has the potential to help rethink how drugs are discovered and evaluated, where scalable molecular measurements across diverse models and within their in vivo context enables us to capture heterogeneity in drug response much earlier in the drug discovery process. Moving beyond simplified biochemical or cell-based assays provides an opportunity to identify drugs not only based on their depth of target engagement but also based on their breadth of activity across many models. Prioritizing this aspect will better ensure that future drugs are more likely to generalize across individuals. Similarly, rich phenotypic data from many tumor models can also allow precise therapies that identify individuals most likely to respond, which in turn leads to more informed selection of participants in subsequent clinical trials. By building a platform like GENEVA, we demonstrate that scalably generating these generalizable, rich datasets can inform an important step toward realizing the promise of precision medicine.

## Methods

### Ethics statement

All experiments were performed in accordance with Institutional Animal Care and Use Committee (IUCAC) and Biological Use Authorization protocols (IUCAC protocols AN201942-00 and AN207247-00A and Institutional Review Board CC 136512 for EML4-ALK and TH21 PDX models).

### Compound sources

Compounds were obtained from MedChemExpress, CaymanChem and SelleckChem. Large batches of ARS-1620 were obtained with thanks from the laboratory of K.M.S.

### Compound treatments

In vitro compound treatments were prepared by diluting the compound to final concentration in complete RPMI supplemented with 10% fetal bovine serum (FBS) and penicillin–streptomycin with no more than 1% DMSO in the final solution. Combination treatments were prepared in the same way. In vitro treatments received medium changes with fresh compound dilutions every 48 h. In vivo delivery was formulated as specified for the specific study and dosed by standard intraperitoneal or oral dosing.

### Cell line sources

All in vitro cell lines were obtained from ATCC, specifically H23 (CRL-5800, male), H358 (CRL-5807, male), H1299 (CRL-5803, male), H1975 (CRL-5908, female), A549 (CCL-185, male), H1792 (CRL-5895, male), H1373 (CRL-5866, male), CALU-1 (HTB-54, male), H441 (HTB-174, male), H2030 (CRL-5914, male), H2122 (CRL-5985, female), SW1573 (CRL-2170, female), SK-LU-1 (HTB-57, female), SK-MEL-2 (HTB-68, male), MeWo (HTB-65, male), HT144 (HTB-63, male), A375 (CRL-1619, female), SK-MEL-28 (HTB-72, male) and MIA PaCa-2 (CRL-1420, male). JAX233 and JAX877 were obtained from The Jackson Laboratories. TH21 and EML4-ALK PDXs were provided by the laboratory of T. Bivona (University of California, San Francisco (UCSF)).

### Organoid culture

PDX tumors were thawed from snap-frozen vials in 10% DMSO and 90% FBS and digested by fine dicing with a razor blade and incubating for 1 h at 37 °C with 1 mg ml^−1^ Liberase. Cell suspensions were strained using 100-μm filters, and resulting cell suspensions were seeded in 100% Matrigel at 37 °C on a dry sterile tissue culture plate. After 30 min, organoid medium was added. Cells were grown for 5 days with daily medium changes, digested using Liberase as described earlier and passaged for expansion or assays. Organoid medium consisted of Advanced DMEM/F12, 1× N-2, 1× B27, 10 mM HEPES buffer, 1× L-glutamine (2 mM) and 1× penicillin–streptomycin (5,000 U ml^−1^)^49^.

### Cell culture and collection

Cell lines were grown in complete RPMI supplemented with 10% FBS and 1× penicillin–streptomycin. Cells were prepared for scRNA-seq by trypsin digestion for 10 min at 37 °C when collected from in vitro culture. For collection of organoids and in vivo xenograft tumors, cells were collected by resection, chopped with a razor blade into a slurry and resuspended in 1 mg ml^−1^ Liberase. Suspensions were incubated with shaking at 37 °C and 150 r.p.m. for 45 min, after which cells were resuspended in ACK buffer and incubated at room temperature for 10 min. Resuspension in 1× PBS was performed two times to wash and remove digestion buffers. Cells were passed through a 70-μm mesh filter to generate single-cell suspensions.

### Creation of GENEVA pools

Cells were seeded in six-well plates at 50,000 cells per well for a *t*_0_ time point. After 72 h, cells were collected by trypsin and counted to determine total cell numbers. Growth rates were calculated using the following formula: *r* = ln (*Y*_*t*_ / *Y*_0_) / *t*. Cell line pooling for GENEVA was performed by collecting all cell lines simultaneously by trypsin digestion, washing in 1× PBS, counting and inversely balancing against growth rate. Cells were seeded at specific numbers inverse to the growth rate into a single sterile tube and mixed well before plating into separate wells in vitro or injected into different mice for flank xenograft studies. All cell line pooling was performed in less than 2 h total time to ensure healthy cell pools. For GENEVA assays in organoid format, mixed cultures were seeded in 100% Matrigel. GENEVA assays in mouse xenografts were mixed in 1:1 PBS:Matrigel at 4 °C before injection into mouse flanks.

### Cell suspensions and cell hashing

Cell suspensions were then hashed using MULTI-seq lipid oligonucleotides or TotalSeq-A antibodies. Cell suspensions were assigned unique lipid-hashtag oligonucleotides (lipid-HTOs) or antibody-HTOs at the point of experimentation. Cells were incubated with corresponding HTO conjugate for 20 min on ice. Cells were then washed three times in 1× cold PBS and recounted. Cells were then pooled together from multiple hashed suspensions such that there were equivalent numbers of cells from each suspension before 10× single-cell preparation. All single-cell studies were hashed in this way.

### GENEVA single-cell library preparation and sequencing

GENEVA libraries were prepared according to scRNA-seq Chromium kit instructions. Modifications were performed after the cDNA step in which the small fractions were collected and amplified in separate PCR reactions for enrichment of the hashtag reads, also as specified in the Chromium kit. For amplification of oligo-HTOs from cDNA libraries, protocols were followed from McGinnis et al.^15^. Sequencing was performed for the transcriptome and hashtag libraries at a 95%/5% ratio (transcriptome/hashtag). Sequencing targets were 25,000 reads per cell based on expected cell counts determined by the number of cells loaded at input into the Chromium kit.

### Data preprocessing

Raw single-cell FASTQ files were used as input into the 10x Cell Ranger pipeline (v3.1.0) with default parameters. Briefly, reads were aligned to the hg38 transcriptome using STAR, generating a filtered feature barcode matrix and BAM alignment file.

### Single-nucleotide polymorphism detection and cell line identification

QuantSeq 3′ RNA-seq FASTQ files for each cell line were first processed using cutadapt (v1.16) to remove adapter sequences. Processed 3′ sequencing reads were then aligned to the hg38 transcriptome using STAR (v2.5.1b). BAM files were then sorted and deduplicated using UMI-tools dedup and used as inputs for variant calling using bcftools mpileup and bcftools call with default parameters. VCF files across all cell lines were merged using bcftools merge.

Pooled scRNA-seq experiments were demultiplexed into individual cell lines using Demuxlet with the CB tag, setting the fixed mixing proportion, *α*, to 0.5. Demultiplexing of single-cell barcodes into drug treatments using HTOs was performed using pymulti from the scEasyMode Python library (https://github.com/johnnyUCSF/scEasyMode). In cases where variant calling with Demuxlet did not yield high enough accuracy, Freemuxlet with a fixed amount of cell clusters corresponding to the amount of cell cluster input was used. Assignment to ground truth cell lines was performed in this case by correlating a pseudobulk cluster from the Freemuxlet group of reads with ground truth individual VCF entries per cell line.

### Postprocessing, doublet removal, quality checks and scoring

Demultiplexed scRNA-seq count matrices were processed using scanpy. Counts were normalized (normalize_per_cell) and log transformed (log1p). Additionally, droplets predicted as doublets by demuxlet were removed. Normalized count matrices were used to calculate principal component analysis, Leiden clusters and UMAP representations. Cells with less than 1,000 uniquely expressed genes (n_genes < 1,000), greater than 15% mitochondrial fraction (mito_frac > 0.15) and less than 2,000 counts (n_counts < 2,000) were identified as apoptotic cells based on thresholds determined by ref. ^50^.

### Differential expression analysis

The scanpy differential expression framework (Wilcoxon rank-sum test) was used to obtain log_2 _(fold change) values between drug and control conditions; log_2_ (fold change) values were then *z*-score normalized across cell lines and averaged to retrieve mean difference *z*scores. Two-tailed *t*-tests for each gene were performed against a bootstrapped distribution of mean difference *z* scores.

G12C-specific expression was determined by first calculating differential expression between DMSO and ARS-1620 treatment for each cell line and then taking the mean log_2_ (fold change) difference between cell lines with G12C and non-G12C mutations. *P* values were calculated using two-tailed *t*-tests between G12C and non-G12C groups.

### Enrichment analysis

iPAGE was used to conduct enrichment analyses using an information-theoretic framework as described in ref. ^51^. Briefly, continuous expression vectors associated with every cell or gene, that is, mitochondrial gene expression (Fig. 3a) or differential expression log_2_ (fold change) values (Extended Data Fig. 6d), were quantized into bins. Binary profile vectors were then created, indicating the presence or absence of drug treatment or genes belonging to a geneset, in each of the bins. iPAGE tests for the mutual information between these vectors were performed and quantified over- and under-representation by modeling a hypergeometric distribution.

### Drug combination analysis

Drug combination efficacy, as measured by Bliss synergy scores, was evaluated by the Bliss independence model as described previously in ref. ^33^. To obtain synergy covariates, first, DESeq2 (ref. ^52^) was used to obtain gene-level linear model coefficients (*β*) using a cell line and drug treatment (single- and dual-) factor design and un-normalized scRNA-seq counts as inputs. For a given cell line, a gene’s synergy covariate was calculated as the difference between the sum of the coefficients derived from the single-drug models, that is, additive effect, and the coefficient of the dual-drug model. Formally, (Ai + Bi)–AB, for drug A and drug B, in a given cell line. The average gene synergy covariate of a gene across all cell lines was taken and ranked based on *t*-statistic values generated from two-tailed *t*-test comparisons to a bootstrapped distribution.

### Gini index transcriptome quantification

To quantify this, we compared the relative abundance of cells from ARS-1620-treated and DMSO control samples within each of the 11 Leiden clusters for this cell line. We observed that ARS-1620-treated cells largely reside in the neighboring clusters ‘11’ and ‘14’. We used the Gini coefficient to compare the unequal distribution of relative cell counts across the gene expression clusters. We then compared the observed Gini index to the null distribution of Gini coefficients calculated for randomly sampled clusters of equal size.

### Survival assays

Cells were seeded in black, clear-bottom 96-well plates at 5,000 cells per well. Cells were allowed to adhere overnight, and the next morning cells were treated with compounds at the specified concentrations in 100 μl of drug medium after removal of seeding medium. After 72 h, cells were lysed by CTG protocol for luminescence reading or incubated for 30 min in Calcein-AM, after which fluorescence reading at FITC channels was performed for data collection.

### Respiration assays

After acute treatment of cells for 2 h with AMG510, cells were immediately processed for standard Seahorse respirometer assays using the Seahorse XF Cell Mito Stress Test protocol detailed at https://www.agilent.com/cs/library/usermanuals/public/XF_Cell_Mito_Stress_Test_Kit_User_Guide.pdf.

### Combination therapies in vitro and in vivo

In vitro compound treatments were prepared by diluting the compound to final concentration in 1× RPMI complete, 10% FBS and penicillin–streptomycin with no more than 1% DMSO in the final solution. In vitro treatments received medium changes with fresh compound dilutions every 48 h. In vivo delivery was formulated as specified for the specific study and dosed by standard intraperitoneal or oral dosing. Note that compounds were prepared as a single suspension for combinations in vivo and dosed as one daily treatment.

### Mitochondrial membrane potential assay

Mitochondrial membrane potential was measured by incubating TMRE at 1 μM for 30 min at 37 °C in base RPMI medium. Cells were then washed twice in 1× PBS and trypsinized for readout by flow cytometry. Detection was performed at 488-nm/575-nm excitation/emission.

### Mitochondrial ROS formation assay

Mitochondrial ROS was measured by incubating MitoSOX Red (Thermo Fisher, M36008) at 0.5 μM for 30 min at 37 °C in RPMI base medium. Cells were then washed twice in 1× PBS and trypsinized for readout by flow cytometry. Detection was performed at 488-nm/575-nm excitation/emission.

### Caspase activation assay

Caspase activation was measured by incubating caspase-3/caspase-7 detection reagent (Thermo Fisher, C10423) at 1 μM for 30 min at 37 °C in RPMI base medium. Cells were then washed twice in 1× PBS and trypsinized for readout by flow cytometry. Detection was performed at 488-nm/575-nm excitation/emission.

### Lipid peroxidation assay

Lipid peroxidation was measured by incubating BODIPY 581/591 C11 detection reagent (Thermo Fisher, C10423) at 10 μM for 30 min at 37 °C in RPMI base medium. Cells were then washed twice in 1× PBS and trypsinized for readout by flow cytometry. Detection was performed at 488-nm/575-nm excitation/emission.

### Animal studies

Animal studies were performed using NSG mice obtained from The Jackson Laboratories. All mice were 8- to 12-week-old females at the point of implantation. Maximal tumor size allowed was 1.5 cm in diameter, and this was not exceeded. Mice were housed under standard controlled conditions on a 12-h light/12-h dark cycle, with temperature maintained at 20–26 °C and humidity between 30 and 70%. Animals were housed at the UCSF Helen Diller Preclinical Tumor Core for all experimentation in accordance with IACUC protocols.

### Xenografts and tumor measurement

Tumors were injected subcutaneously at 100 μl in each flank in a 1:1 ratio of 1× PBS to Matrigel. Cell suspensions were prepared at 20 million cells per ml and kept on ice during and before injection. Tumor measurements were performed by 3D caliper measurements.

### In vivo drug treatment

In vivo drug treatment was performed by diluting compound in 100% DMSO and dilution of the compound by subsequent addition of Labrasol and distilled water for a final formulation of 10% DMSO, 70% Labrasol and 20% water. Dosing for all studies was performed by oral gavage at 100 μl with 5 ON/2 OFF daily dosing. Antimycin was separately dosed in 20% DMSO and 80% distilled water at 50 μl by intraperitoneal injection with 5 ON/2 OFF daily dosing.

### Cell line engineering

HEK293T cells were maintained in complete RPMI and 10% FBS with 1× penicillin–streptomycin and seeded at 2 × 10^6^ cells per 15-cm dish 24 h before transfection. Cells were transfected using *Trans*IT-Lenti Transfection Reagent (MirusBio, 6604) with dCas9-KRAB transfer plasmid, pCMV-dR8.91 and pMD2.G (Addgene, 12259). Medium was changed 16 h after transfection, with new medium supplemented with ViralBoost Reagent (Alstem, VB100). Virus-containing supernatant was collected 48 h after transfection and filtered through a 0.45-μm filter. Functional titer for the target cell lines was performed with serial dilution of virus, and flow cytometry was used as a readout 48 h after transduction. Based on the titer, a viral dilution corresponding to 30–50% transduction efficiency was used for full-scale transduction. Target cell lines were seeded at 9 × 10^5^ cells per 10-cm dish 24 h before transduction, and medium was replaced 24 h after transduction. Transduced cells were grown and sorted for mCherry expression, a process that was repeated once more for purity.

### Pooled guide cloning

Pooled dual sgRNA libraries were cloned in general accordance with Protocol 1 from the laboratory of J.W. (https://weissman.wi.mit.edu/resources/) using the lentiviral vector pJR85 (Addgene, 140095) and drop-in from pJR89 (Addgene, 140096), and an oligonucleotide pool of dual sgRNAs was ordered from Twist Biosciences. FastDigest BstXI (Thermo Fisher, FD1024) and FastDigest Bpu1102I (Thermo Fisher, FD0094) were used for the digestion of pJR85 and oligonucleotide pool for 4 h at 37 °C. Balanced and complete sgRNA representation of the final plasmid pool was confirmed via Illumina Miseq, and lentivirus was generated using HEK293T cells transfected with *Trans*IT-Lenti Transfection Reagent (MirusBio, 6604), the sgRNA plasmid pool, pCMV-dR8.91 and pMD2.G (Addgene, 12259).

### Dosing and sample collection

For in vivo dual-guide CRISPRi screens, 1.5 million cancer cells were resuspended in 100 μl of 1:1 PBS:Matrigel and injected into both flanks of 9-week-old female NSG mice. Sotorasib was solubilized in 10% (vol/vol) DMSO + 90% (vol/vol) of 20% (wt/vol) Captisol (Ligand Pharmaceuticals) in saline. Two days after implantation, daily oral gavage of 100 μl of either sotorasib (100 mg per kg (body weight)) or vehicle was administered for 13 days. Mice were killed, tumors were excised on day 14, and genomic DNA was extracted using a NucleoSpin Blood XL kit (Macherey-Nagel, 740950.50). For in vitro dual-guide CRISPRi screens, 200,000 cells were seeded into each well of a six-well plate, and RPMI with either sotorasib solubilized in DMSO (30 nM) or 0.1% DMSO was administered every 2–3 days for 12 days. Cells were collected on day 12, and genomic DNA was extracted using a NucleoSpin Blood XL kit (Macherey-Nagel, 740950.50).

### gRNA library preparation and sequencing

gRNA libraries were prepared in general accordance with Protocol 2 from the laboratory of J.W. (https://weissman.wi.mit.edu/resources/), and sequencing was performed using an Illumina NovaSeq X (R1: 19, I1:8, R2: 19, I2: 8).

### Guide scoring analysis

We fit a binomial (logit) model to compare sgRNA counts under different treatments and genetic backgrounds, weighting by total reads. Resulting *z* scores were normalized via bestNormalize and aggregated per gene to yield a combined *z* score. Genes were ranked by this metric, and significance was determined after Benjamini–Hochberg correction. Annotations used known ‘Sensitizing’ or ‘Protective’ classifications from GENEVA predictions.

### Gene annotation from GENEVA predictions

Scoring was performed based on the direction of effect observed based on (1) GENEVA data indicating upregulation/downregulation after KRAS-G12C inhibitor treatment from experiments in Figs. 1–4 and (2) the contribution of that gene to the pathway biology (suppressor versus effector) from the literature. Full gene-level annotations can be found within the Supplementary Information.

### Statistics and reproducibility

No statistical methods were used to predetermine sample sizes for in vivo experiments, but we used a published similar article to determine study size^53^. For experimental replicates, all dose–response plate-based assays were run at three or more replicates per data point. For Seahorse assays, each data point consisted of ten replicates. For single-cell experiments, each experiment contained 10,000 or more cells based on feasibility and cost. For flow cytometry assays, a minimum of two wells and thousands of cells per well were collected for each data point, with bootstrapping used to sample without replacement data from both sets of cells. Data distribution was assumed to be normal, but this was not formally tested. No animals were excluded from the analyses. Data exclusion criteria applied only to single-cell GENEVA data where certain cell lines did not yield a sufficient number of cells (<1% of the population). This led to low power for drug responses for those cell lines and therefore were excluded because they were too low to accurately be measured. Data collection and analysis were not performed blind to the conditions of the experiments. Mice were allocated randomly from the same litter age into equivalent groups before implantation and treatment.

### Reporting summary

Further information on research design is available in the Nature Portfolio Reporting Summary linked to this article.

## Supplementary information

Supplementary Information Supplementary Information and Supplementary Fig. 1.

Reporting Summary

## Source data

Source Data Fig. 1 Statistical source data, with tabs for items.

Source Data Fig. 2 Statistical source data, with tabs for items.

Source Data Fig. 3 Statistical source data, with tabs for items.

Source Data Fig. 4 Statistical source data, with tabs for items.

Source Data Fig. 5 Statistical source data, with tabs for items.

Source Data Extended Data Fig. 1 Statistical source data, with tabs for items.

Source Data Extended Data Fig. 2 Statistical source data, with tabs for items.

Source Data Extended Data Fig. 3 Statistical source data, with tabs for items.

Source Data Extended Data Fig. 4 Statistical source data, with tabs for items.

Source Data Extended Data Fig. 5 Statistical source data, with tabs for items.

Source Data Extended Data Fig. 6 Statistical source data, with tabs for items.

Source Data Extended Data Fig. 7 Statistical source data, with tabs for items.

Source Data Extended Data Fig. 8 Statistical source data, with tabs for items.

## Data Availability

The sequencing datasets generated in this study have been deposited in the Gene Expression Omnibus under the accession number GSE283335. Meta-analysis of synthetic lethal CRISPR screen data for different compounds consisted of six publicly available datasets^21,24–26,54,55^. Further information on research design is available in the Nature Research Reporting Summary linked to this article. Source data are provided with this paper. All other data supporting the findings of this study are available from the corresponding author on reasonable request.

## Code availability

Code are publicly available at https://github.com/goodarzilab/GENEVA.
