# Supplementary material for: The GENEVA platform models tumor mosaicism to reveal variations of responses to KRAS inhibitors and identify improved drug combinations
Source: Nat Cancer. 2026 Feb 24;7(3):522–37. doi: 10.1038/s43018-026-01130-5 (PMC13035475; doi:10.1038/s43018-026-01130-5)
Supplement: Supplementary file 1 — Supplementary Information and Supplementary Fig. 1. [file 43018_2026_1130_MOESM1_ESM.pdf]

nature cancer

Supplementary information https://doi.org/10.1038/s43018-026-01130-5

The GENEVA platform models tumor mosaicism to reveal variations of responses to KRAS inhibitors and identify improved drug combinations

In the format provided by the authors and unedited

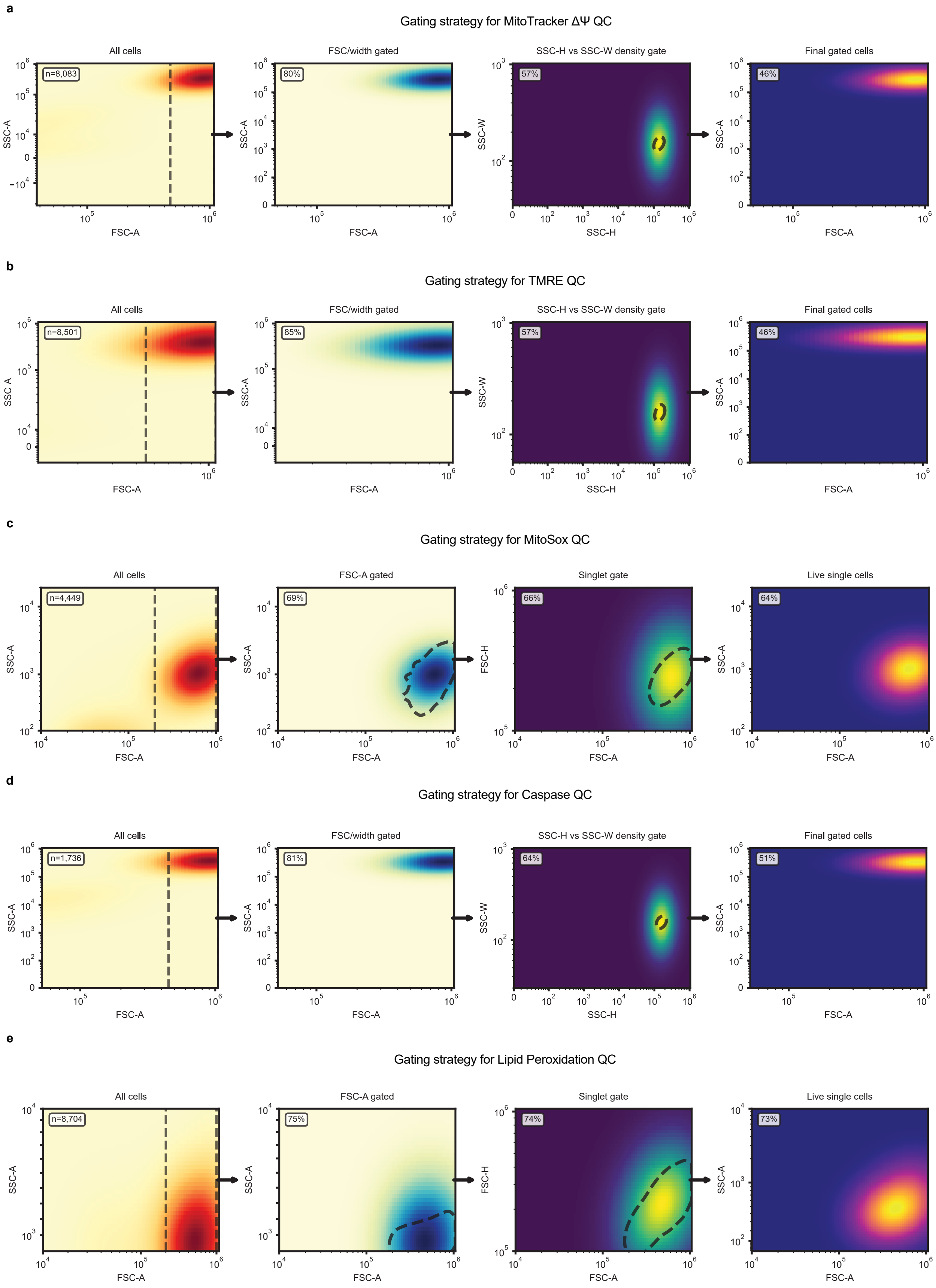

Supplementary Figure 1. (a) Flow cytometry gating strategy for mitotracker delta psi values. Shown is a representative sample across all gating strategies used to select for live cells before quantification of mitotracker fluorophores. **(b)** Flow cytometry gating strategy for TMRE values. Shown is a representative sample across all gating strategies used to select for live cells before quantification of TMRE fluorophores. **(c)** Flow cytometry gating strategy for mitosox values. Shown is a representative sample across all gating strategies used to select for live cells before quantification of mitosox fluorophores. **(d)** Flow cytometry gating strategy for caspase cleavage values. Shown is a representative sample across all gating strategies used to select for live cells before quantification of caspase fluorophores. **(e)** Flow cytometry gating strategy for lipid peroxidation values. Shown is a representative sample across all gating strategies used to select for live cells before quantification of lipid peroxidation fluorophores.

guide	seq	relative_direction	notes	pathway	gene_name
MTOR_1_A	GGCAGGGGGCCTGAAGCGG	vehicle	inhibits mTOR signaling	mTOR	MTOR
RPTOR_1_A	ACCCCAGGTCCCAAGCCAC	vehicle	inhibits mTOR signaling	mTOR	RPTOR
MLST8_1_A	GGCCTCCGTACCTTACGGG	vehicle	inhibits mTOR signaling	mTOR	MLST8
RICTOR_1_A	GCGCAGGGCGGAATGACAG	vehicle	inhibits mTOR signaling	mTOR	RICTOR
AKT1_1_A	GCGCGGGCCCGGCCAAGGG	vehicle	inhibits mTOR signaling	mTOR	AKT1
AKT2_1_A	GTATTTCCGTCCAGAGGGT	vehicle	inhibits mTOR signaling	mTOR	AKT2
AKT3_1_A	CCGGCGGGAGGGCAAGCGA	vehicle	inhibits mTOR signaling	mTOR	AKT3
TSC1_1_A	CTGTGAGGTAAACAGCTGA	undetermined	negative regulator of mTORC1	mTOR	TSC1
TSC2_1_A	GGGAACGCAGGGCCGCACG	sotorasib	negative regulator of mTORC1	mTOR	TSC2
DEPTOR_1_A	GCGCGGGAAGCGTCTGTGA	sotorasib	negative regulator of mTORC1	mTOR	DEPTOR
PIK3CA_1_A	CAGCAGCCCGACACCTCAG	vehicle	inhibits mTOR signaling	mTOR	PIK3CA
PIK3CB_1_A	CACACGGGCGCGGGTTCGG	vehicle	inhibits mTOR signaling	mTOR	PIK3CB
PIK3CD_1_A	CCTGCCAGCTGCGCCGGGA	vehicle	inhibits mTOR signaling	mTOR	PIK3CD
PIK3CG_1_A	ATTTAGACGCACACTGGGT	vehicle	inhibits mTOR signaling	mTOR	PIK3CG
RHEB_1_A	CTCCCAGGCGAGGCCCCGG	vehicle	inhibits mTOR signaling	mTOR	RHEB
PRKAA1_1_A	CGTGTCACCCAGAATGTAG	sotorasib	negative regulator of mTORC1	mTOR	PRKAA1
PRKAA2_1_A	AGGCCGCGCGCGCCGAAGA	sotorasib	negative regulator of mTORC1	mTOR	PRKAA2
PPP2CA_1_A	GAGCCTCAGCGAGCGGAGG	sotorasib	negative regulator of mTORC1	mTOR	PPP2CA
PPP2CB_1_A	GCGTGAGGCGGGCCTGAAG	sotorasib	negative regulator of mTORC1	mTOR	PPP2CB
EIF4EBP1_1_A	CACAGGAGACCATGTCCGG	sotorasib	negative regulator of mTORC1	mTOR	EIF4EBP1
RPS6KB1_1_A	CAGCCGCTGCCGCCATCAC	vehicle	inhibits mTOR signaling	mTOR	RPS6KB1
ULK1_1_A	TCCGACTCCGGCTCCAACT	sotorasib	negative regulator of mTORC1	mTOR	ULK1
SNAI1_1_A	CGCGGCACGGCCTAGCGAG	vehicle	inhibitor of EMT process	EMT	SNAI1
SNAI2_1_A	TCAGGTGCGGCAGACGGAC	vehicle	inhibitor of EMT process	EMT	SNAI2
SNAI3_1_A	GACGCAGCAGTCCGGACCC	vehicle	inhibitor of EMT process	EMT	SNAI3
ZEB1_1_A	CAGCGGCAACAGCTCAATA	vehicle	inhibitor of EMT process	EMT	ZEB1
ZEB2_1_A	TCCAGGAAACACAAACCTG	vehicle	inhibitor of EMT process	EMT	ZEB2
TWIST1_1_A	CCGCCAGGCCTCCTGGAAA	vehicle	inhibitor of EMT process	EMT	TWIST1
TWIST2_1_A	CAGCGAGGAGGAGCTCGAG	vehicle	inhibitor of EMT process	EMT	TWIST2
MRPL16_1_A	GGAGCGCCGCTGCTTACCT	sotorasib	rescues sotorasib effect	mRibosome	MRPL16
MRPS21_1_A	GTACTGAGCGCGCGAGGTG	sotorasib	rescues sotorasib effect	mRibosome	MRPS21
MRPS5_1_A	GACTCCAGCATGGCGACCG	sotorasib	rescues sotorasib effect	mRibosome	MRPS5
MRPL30_1_A	CCCTTCGGAGGAAAATTTC	sotorasib	rescues sotorasib effect	mRibosome	MRPL30
MRPL14_1_A	AGGCCGCGTGGGGCGAAGG	sotorasib	rescues sotorasib effect	mRibosome	MRPL14
RBMS3_1_A	GCAGCAACTAAGCTGTACA	vehicle	upregulated in persistor cells, targets for combo therapy	RNA Binding Protein	RBMS3
EHMT2_1_A	CCAGCGCAAGCGGCGATGG	vehicle	upregulated in persistor cells, targets for combo therapy	Histone Methyl Transferase	EHMT2
GPX4_1_A	AGGCGGCCGAGGCTCATCG	vehicle	upregulated in persistor cells, targets for combo therapy	Ferroptosis	GPX4
IDH2_1_A	CTCTCCAGCTTGGGATGGC	vehicle	upregulated in persistor cells, targets for combo therapy	Metabolism	IDH2
VEGFB_1_A	GAGTGCGGCGAGCAGCAGG	vehicle	upregulated in persistor cells, targets for combo therapy	Vascularization	VEGFB
AURKA_1_A	CGCCAAGGACACTCAGGGA	vehicle	upregulated in persistor cells, targets for combo therapy	Mitosis	AURKA
TGFBI_1_A	GAGCGAGCTAGCGACCGAC	undetermined	unclear, could be either way	EMT	TGFBI
PARP14_1_A	CGCGGCCCCTGCAGTCCGG	vehicle	upregulated in persistor cells, targets for combo therapy	DNA Damage Repair	PARP14
JAK1_1_A	GCAGCTCCAGGATACTCCG	vehicle	upregulated in persistor cells, targets for combo therapy	JAK-STAT	JAK1
KRAS_1_A	GGCGCCGGCAAAGAGGGTC	sotorasib	protein target of Sotorasib	RAS Oncogene	KRAS
PTPN11_1_A	GAGGAACATGACATCGCGG	vehicle	SHP2 is syn lethal target as described by the field	Tyrosine Phosphatase (SHP2)	PTPN11
SOS1_1_A	TCCAGCGCTACACCGGCGG	vehicle	SHP2 is syn lethal target as described by the field	RAS GEF	SOS1
ctrl1_1_A	GAGTTAAGGCCTCGTCTAG	ctrl	control	Control	ctrl1
ctrl2_1_A	TCCCAGGCTCTCCACTATG	ctrl	control	Control	ctrl2
ctrl3_1_A	GACGCGTCTGCAAGAACGT	ctrl	control	Control	ctrl3
ctrl4_1_A	GGCATGGACCCGCGGCACG	ctrl	control	Control	ctrl4
ctrl5_1_A	CGTCCGAGGTACTGAATAA	ctrl	control	Control	ctrl5
ctrl6_1_A	TCTGACTCTCCGTCCACCA	ctrl	control	Control	ctrl6
MTOR_2_A	GGACAGCGGGGAAGGCGGG	vehicle	inhibits mTOR signaling	mTOR	MTOR
