# Supplementary material for: The GENEVA platform models tumor mosaicism to reveal variations of responses to KRAS inhibitors and identify improved drug combinations
Source: Nat Cancer. 2026 Feb 24;7(3):522–37. doi: 10.1038/s43018-026-01130-5 (PMC13035475; doi:10.1038/s43018-026-01130-5)
Supplement: Supplementary file 2 — Reporting Summary [file 43018_2026_1130_MOESM2_ESM.pdf]

Corresponding author(s): Hani Goodarzi

Last updated by author(s): Jan 8, 2026

# Reporting Summary

Nature Portfolio wishes to improve the reproducibility of the work that we publish. This form provides structure for consistency and transparency in reporting. For further information on Nature Portfolio policies, see our Editorial Policies and the Editorial Policy Checklist.

## Statistics

For all statistical analyses, confirm that the following items are present in the figure legend, table legend, main text, or Methods section.

| n/a | Confirmed | |
|---|---|---|
| ☐ | ☒ | The exact sample size (*n*) for each experimental group/condition, given as a discrete number and unit of measurement |
| ☐ | ☒ | A statement on whether measurements were taken from distinct samples or whether the same sample was measured repeatedly |
| ☐ | ☒ | The statistical test(s) used AND whether they are one- or two-sided<br>*Only common tests should be described solely by name; describe more complex techniques in the Methods section.* |
| ☐ | ☒ | A description of all covariates tested |
| ☐ | ☒ | A description of any assumptions or corrections, such as tests of normality and adjustment for multiple comparisons |
| ☐ | ☒ | A full description of the statistical parameters including central tendency (e.g. means) or other basic estimates (e.g. regression coefficient) AND variation (e.g. standard deviation) or associated estimates of uncertainty (e.g. confidence intervals) |
| ☐ | ☒ | For null hypothesis testing, the test statistic (e.g. *F*, *t*, *r*) with confidence intervals, effect sizes, degrees of freedom and *P* value noted<br>*Give P values as exact values whenever suitable.* |
| ☐ | ☒ | For Bayesian analysis, information on the choice of priors and Markov chain Monte Carlo settings |
| ☐ | ☒ | For hierarchical and complex designs, identification of the appropriate level for tests and full reporting of outcomes |
| ☐ | ☒ | Estimates of effect sizes (e.g. Cohen's *d*, Pearson's *r*), indicating how they were calculated |

*Our web collection on statistics for biologists contains articles on many of the points above.*

## Software and code

Policy information about availability of computer code

**Data collection**

Plate reader software from Biotek was used to collect plate based luminescence and fluorescence readings. Agilent software was used to collect data from Seahorse instruments. Flow cytometry was performed using software from BD made for the Aria2 and also Attune software for the attune high-throughput flow cytometer. Sequencing was performed with Nextseq, Miseq, and Novaseq using Illumina software for demultiplexing and sequencing. 10X Chromium controller was used for processing single cells and the corresponding chromium software was used to control the fluidics device within the Chromium controller.

**Data analysis**

Code will be available at https://github.com/goodarzilab/GENEVA

Data analysis was performed using the python packages:
zipp==0.6.0
wheel==0.33.4
webencodings==0.5.1
wcwidth==0.1.7
urllib3==1.25.3
umap-learn==0.3.10
traitlets==4.3.3
tornado==5.1.1
testpath==0.4.2
terminado==0.8.2
tables==3.4.4
statsmodels==0.9.0

```
sklearn==0.0
six==1.12.0
setuptools==41.0.1
send2trash==1.5.0
seaborn==0.9.0
scipy==1.2.1
scikit-learn==0.20.2
scanpy==1.4
s3transfer==0.2.1
ruamel-yaml==0.15.46
rsa==3.4.2
requests==2.21.0
pyzmq==18.1.0
pyyaml==5.1
pytz==2018.9
python-levenshtein==0.12.0
python-igraph==0.7.1.post6
python-dateutil==2.8.0
pysocks==1.7.0
pyrsistent==0.15.4
pyparsing==2.3.1
pyopenssl==19.0.0
pygments==2.4.2
pycparser==2.19
pycosat==0.6.3
pycairo==1.18.0
pyasn1==0.4.6
ptyprocess==0.6.0
prompt-toolkit==2.0.10
prometheus-client==0.7.1
pip==19.2.1
pickleshare==0.7.5
pexpect==4.7.0
patsy==0.5.1
parso==0.5.1
pandocfilters==1.4.2
pandas==0.24.1
numpy==1.15.4
numexpr==2.6.9
numba==0.42.0
notebook==6.0.1
networkx==2.2
nbformat==4.4.0
nbconvert==5.6.0
natsort==6.0.0
more-itertools==7.2.0
mkl-random==1.0.2
mkl-fft==1.0.10
mistune==0.8.4
matplotlib==3.0.2
markupsafe==1.1.1
louvain==0.6.1
llvmlite==0.27.0
kiwisolver==1.0.1
jupyterlab==1.1.4
jupyterlab-server==1.0.6
jupyter-core==4.5.0
jupyter-client==5.3.3
jsonschema==3.1.1
json5==0.8.5
joblib==0.13.2
jmespath==0.9.4
jinja2==2.10.3
jedi==0.15.1
ipython==7.8.0
ipython-genutils==0.2.0
ipykernel==5.1.3
importlib-metadata==0.23
idna==2.8
h5py==2.9.0
fuzzywuzzy==0.17.0
fastcluster==1.1.25
entrypoints==0.3
docutils==0.14
defusedxml==0.6.0
decorator==4.4.0
```

cython==0.29.14
cycler==0.10.0
cryptography==2.7
conda==4.6.7
colorama==0.3.9
chardet==3.0.4
cffi==1.12.3
certifi==2020.12.5
botocore==1.12.200
bleach==3.1.0
biopython==1.72
bbknn==1.3.6
backcall==0.1.0
awscli==1.16.210
attrs==19.3.0
asn1crypto==0.24.0
annoy==1.16.2
anndata==0.6.18
flowcal==1.3.0
synergy==0.5.1

For manuscripts utilizing custom algorithms or software that are central to the research but not yet described in published literature, software must be made available to editors and reviewers. We strongly encourage code deposition in a community repository (e.g. GitHub). See the Nature Portfolio guidelines for submitting code & software for further information.

## Data

Policy information about availability of data

All manuscripts must include a data availability statement. This statement should provide the following information, where applicable:

- Accession codes, unique identifiers, or web links for publicly available datasets
- A description of any restrictions on data availability
- For clinical datasets or third party data, please ensure that the statement adheres to our policy

The sequencing datasets generated in this study have been deposited in the Gene Expression Omnibus (GEO) under the accession number GSE283335.
Meta-analysis of synthetic lethal CRISPR screen data for different compounds come from the following publicly available data:
https://pubmed.ncbi.nlm.nih.gov/28985505/,
https://www.science.org/doi/full/10.1126/science.abl5829, https://www.ncbi.nlm.nih.gov/pmc/articles/PMC6104643/?report=reader#!po=1.72414, https://journals.plos.org/plosbiology/article?id=10.1371/journal.pbio.2004624#sec039, https://journals.plos.org/plosgenetics/article?id=10.1371/journal.pgen.1008057#sec034, https://pubmed.ncbi.nlm.nih.gov/31138768/
Source data for Fig. 1-5 and Extended Data Fig. 1-6 have been provided as Source Data files. All other data supporting the findings of this study are available from the corresponding author on reasonable request.

## Human research participants

Policy information about studies involving human research participants and Sex and Gender in Research.

| | |
|---|---|
| Reporting on sex and gender | *Use the terms sex (biological attribute) and gender (shaped by social and cultural circumstances) carefully in order to avoid confusing both terms. Indicate if findings apply to only one sex or gender; describe whether sex and gender were considered in study design whether sex and/or gender was determined based on self-reporting or assigned and methods used. Provide in the source data disaggregated sex and gender data where this information has been collected, and consent has been obtained for sharing of individual-level data; provide overall numbers in this Reporting Summary. Please state if this information has not been collected. Report sex- and gender-based analyses where performed, justify reasons for lack of sex- and gender-based analysis.* |
| Population characteristics | *Describe the covariate-relevant population characteristics of the human research participants (e.g. age, genotypic information, past and current diagnosis and treatment categories). If you filled out the behavioural & social sciences study design questions and have nothing to add here, write "See above."* |
| Recruitment | *Describe how participants were recruited. Outline any potential self-selection bias or other biases that may be present and how these are likely to impact results.* |
| Ethics oversight | *Identify the organization(s) that approved the study protocol.* |

Note that full information on the approval of the study protocol must also be provided in the manuscript.

# Field-specific reporting

Please select the one below that is the best fit for your research. If you are not sure, read the appropriate sections before making your selection.

☒ Life sciences ☐ Behavioural & social sciences ☐ Ecological, evolutionary & environmental sciences

For a reference copy of the document with all sections, see nature.com/documents/nr-reporting-summary-flat.pdf

# Life sciences study design

All studies must disclose on these points even when the disclosure is negative.

| | |
|---|---|
| Sample size | No sample size calculations were performed. For experimental replicates, all dose-response plate based assays were run at 3+ replicates per data point. For seahorse assays, each data point had 10 replicates. For single cell experiments, each experiment contained 10,000+ cells based on feasibility and cost. For flow cytometry assays, a minimum of two wells and thousands of cells per well were collected for each datapoint with bootstrapping used to sample without replacement data from both sets of cells. |
| Data exclusions | Data exclusion criteria applied only to single-cell GENEVA data where certain cell lines did not yield a sufficient number of cells (<1% of the population). This led to low power for drug responses for those cell lines and therefore were excluded because they were too low to accurately be measured. |
| Replication | Experiments were performed initially at lower scale for assay optimization. Once technical measurement capabilities were established we conducted the experiment at higher throughput and at the correct conditions for detection. Single-cell experiments were repeated in different study designs to confirm the robustness of GENEVA. This was done over 5 experiments comprising different models (Cell lines, organoids, CDX, PDX) and at different composition of pools from the perspective of multiple models. |
| Randomization | Mice were allocated randomly from the same litter age into equivalent groups before implantation and treatment. |
| Blinding | Due to material and labor constraints, the mouse work was not able to be randomized throughout the collection process for all studies in terms of separating data collection by caliper measurements from the identity of the cohort. |

# Reporting for specific materials, systems and methods

We require information from authors about some types of materials, experimental systems and methods used in many studies. Here, indicate whether each material, system or method listed is relevant to your study. If you are not sure if a list item applies to your research, read the appropriate section before selecting a response.

## Materials & experimental systems

| n/a | Involved in the study | |
|---|---|---|
| ☒ | ☐ | Antibodies |
| ☐ | ☒ | Eukaryotic cell lines |
| ☒ | ☐ | Palaeontology and archaeology |
| ☐ | ☒ | Animals and other organisms |
| ☒ | ☐ | Clinical data |
| ☒ | ☐ | Dual use research of concern |

## Methods

| n/a | Involved in the study | |
|---|---|---|
| ☒ | ☐ | ChIP-seq |
| ☐ | ☒ | Flow cytometry |
| ☒ | ☐ | MRI-based neuroimaging |

## Eukaryotic cell lines

Policy information about cell lines and Sex and Gender in Research

| | |
|---|---|
| Cell line source(s) | All cell lines were derived from ATCC prior to passaging and utilization in experiments. H23 (ATCC CRL-5800, male); H358 (ATCC CRL-5807, male); H1299 (ATCC CRL-5803, male); H1975 (ATCC CRL-5908, female); A549 (ATCC CCL-185, male); H1792 (ATCC CRL-5895, male); H1373 (ATCC CRL-5866, male); Calu-1 (ATCC HTB-54, male); H441 (ATCC HTB-174, male); H2030 (ATCC CRL-5914, male); H2122 (ATCC CRL-5985, female); SW1573 (ATCC CRL-2170, female); SK-LU-1 (ATCC HTB-57, female); SK-MEL-2 (ATCC HTB-68, male); MeWo (ATCC HTB-65, male); HT144 (ATCC HTB-63, male); A375 (ATCC CRL-1619, female); SK-MEL-28 (ATCC HTB-72, male); MIA PaCa-2 (ATCC CRL-1420, male). |
| Authentication | Cell line authentication was not performed for the banks used in these studies. |
| Mycoplasma contamination | Mycoplasma testing was conducted every third monthl throughout the course of experiments. |
| Commonly misidentified lines (See ICLAC register) | None were used. |

# Animals and other research organisms

Policy information about studies involving animals; ARRIVE guidelines recommended for reporting animal research, and Sex and Gender in Research

| | |
|---|---|
| Laboratory animals | Animal studies were performed using NOD.Cg-Prkdcscid Il2rgtm1Wjl/SzJ (NSG) mice obtained from The Jackson laboratories. All mice were 8-12 week old females at the point of implantation. Mice were housed under standard controlled conditions with a 12:12 hour light:dark cycle, temperature maintained at 68-79°F (20-26°C), and humidity between 30-70%." |
| Wild animals | No wild animals were used. |
| Reporting on sex | Female mice were only used in these studies. |
| Field-collected samples | No field-collected samples were used. |
| Ethics oversight | IACUC approval was granted via UCSF LARC and IACUC boards. . IACUC Protocols #AN201942-00 and #AN207247-00A, and IRB CC #136512 for EML4-ALK and TH21 PDX models. Maximal tumor size allowed was 1.5cm in diameter, and this was not exceeded. |

Note that full information on the approval of the study protocol must also be provided in the manuscript.

# Flow Cytometry

## Plots

Confirm that:

☒ The axis labels state the marker and fluorochrome used (e.g. CD4-FITC).

☒ The axis scales are clearly visible. Include numbers along axes only for bottom left plot of group (a 'group' is an analysis of identical markers).

☒ All plots are contour plots with outliers or pseudocolor plots.

☒ A numerical value for number of cells or percentage (with statistics) is provided.

## Methodology

| | |
|---|---|
| Sample preparation | Cells were treated in multi-well plates and treated prior to flow cytometery. Adherent cells were trypsinized before direct labeling using markers of mitochondrial content, mitochondrial ROS, lipid peroxidation, mitochondrial membrane potential, and before injection for flow cytometry. |
| Instrument | BD ARIA2, Attune Nxt |
| Software | BD FACSDIVA, Attune Nxt |
| Cell population abundance | *Describe the abundance of the relevant cell populations within post-sort fractions, providing details on the purity of the samples and how it was determined.* |
| Gating strategy | FSC/SSC gating was performed to remove dead cells. Gating was performed by identifying FSC/SSC high cluster and drawing a polygonal gate around this population to exclude doublets and debris. |

☒ Tick this box to confirm that a figure exemplifying the gating strategy is provided in the Supplementary Information.
